# Seasonal Stratification Drives Bioaccumulation of Pelagic Mercury Sources in Eutrophic Lakes

**DOI:** 10.1021/acsestwater.5c00028

**Published:** 2025-04-10

**Authors:** Grace J. Armstrong, Sarah E. Janssen, Ryan F. Lepak, Tylor J. Rosera, Benjamin D. Peterson, Samia T. Cushing, Michael T. Tate, James P. Hurley

**Affiliations:** † U.S. Geological Survey, Upper Midwest Water Science Center, Madison, Wisconsin 53726, United States; ‡ Environmental Chemistry and Technology Program, University of Wisconsin-Madison, Madison, Wisconsin 53706, United States; § U.S. EPA Office of Research and Development, Center for Computational Toxicology and Exposure, Great Lakes Ecology and Toxicology Division, 6201 Congdon Blvd., Duluth, Minnesota 55804, United States; ∥ Department of Bacteriology, University of Wisconsin−Madison, Madison, Wisconsin 53706, United States; ⊥ Department of Environmental Toxicology, University of California-Davis, Davis, California 95616 United States; # Freshwater@UW Summer Research Opportunities Program, University of Wisconsin-Madison, Madison, Wisconsin 53706, United States; ∇ University of Wisconsin Aquatic Sciences Center, Madison, Wisconsin 53706, United States

**Keywords:** mercury, methylmercury, eutrophication, freshwater, bioaccumulation, stable isotopes

## Abstract

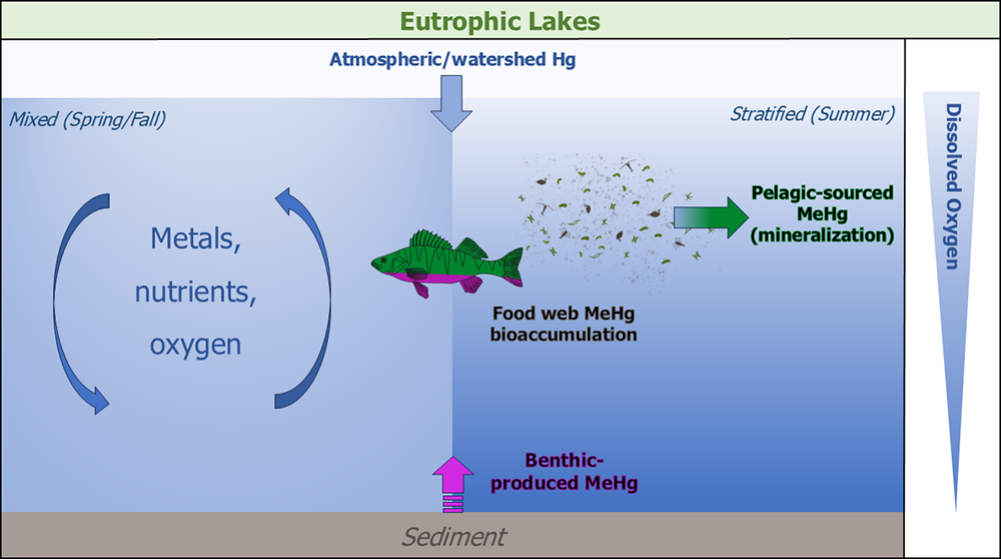

Increased lake eutrophication, influenced by changing climate and land use, alters aquatic cycling and bioaccumulation of mercury (Hg). Additionally, seasonally dynamic lake circulation and plankton community composition can confound our ability to predict changes in biological Hg concentrations and sources. To assess temporal variation, we examined seasonal total Hg (THg) and methylmercury (MeHg) concentrations and stable isotope values in seston, waters, sediments, and fish from two adjacent urban eutrophic lakes in Madison, Wisconsin. In Lake Monona, surface sediment THg concentrations were elevated due to comparably higher urbanization and historical industrial inputs, whereas Lake Mendota sediments had lower concentrations corresponding with largely agricultural and suburban surrounding watershed. Surface water THg and MeHg were similar between lakes and seasonally dynamic, but water profiles exhibited elevated concentrations in the meta- and hypolimnia, highlighting water column MeHg production. Seston MeHg concentrations were often highest at shoulder seasons, possibly owing to metalimnetic MeHg delivery, but also differences in biomass and water clarity. The Δ^199^Hg and δ^202^Hg values in seston were similar between lakes, despite differing sediment THg concentrations and isotope values, suggesting a shared bioaccumulated source of MeHg. Measurement of MeHg stable isotopes further elucidated that seston and fish predominantly bioaccumulated pelagic-sourced MeHg.

## Abstract 2

Mercury species concentrations and stable isotope values reveal that eutrophic lakes display unique seasonally dynamic Hg cycling within the water column and food web.

## Introduction

Mercury (Hg) is a persistent contaminant of global concern due to its long-range transport in the atmosphere, high propensity to bioaccumulate as methylmercury (MeHg), and role as a potent neurotoxin.^1^ In aquatic environments, inorganic Hg (iHg) can be converted into MeHg by microorganisms through the process of methylation.^2^ MeHg enters aquatic food webs primarily by way of uptake into basal resources (e.g., phytoplankton) and subsequently undergoes biomagnification through trophic transfer,^3^ resulting in elevated concentrations and numerous fish consumption advisories. MeHg burdens and bioaccumulation factors within lakes are driven by various factors like Hg source (e.g., legacy point source or nonpoint source runoff, termed watershed Hg), delivery pathway (e.g., dry deposition, wet precipitation, direct discharge),^4^ biogeochemical parameters (e.g., pH, dissolved organic matter; DOM),^5−7^ ecological factors (e.g., trophic status, plankton size),^5,8,9^ and physical lake cycling dynamics.^10^ In particular, it has been difficult to elucidate Hg sources in sites with mixed source contributions (e.g., legacy, watershed, atmospheric deposition), often an issue for waterbodies in or adjacent to urban development.^11^ Efforts by resource managers to further mitigate Hg contamination have been hindered by a lack of understanding regarding the availability of different Hg sources within ecosystems, which are everchanging due to co-occurring stressors like eutrophication.^12^

According to a national-scale survey, lakes in the United States are becoming increasingly eutrophic due to human activities and climate change, with 73.4 ± 9.8% of lakes (>1 ha in total size) being categorized as eutrophic or hypereutrophic on average in 2022, a 16.1% increase from 2012,^13^ highlighting the need to further improve our understanding of the effects of eutrophication on Hg cycling. Eutrophication has complex impacts on food web Hg concentrations in freshwater and marine aquatic systems. Excessive nutrient loads (e.g., nitrogen and phosphorus) in eutrophic lakes can increase Hg methylation and bioaccumulation by stimulating algal growth, leading to enhanced oxygen depletion (i.e., hypoxia) in the water column, higher rates of organic carbon settling, and less sunlight penetration.^14,15^ These changes can be particularly pronounced within eutrophic dimictic (mixing twice a year) lakes that are influenced by annual circulation patterns, driven by differences in temperature density gradients, that result in seasonal shifts in water column biogeochemistry and changes in algal community composition.^16−18^ Eutrophic lake mixing not only affects distribution of Hg species in the water column but also the rate of production of MeHg.^19^ Specific water column parameters such as redox status, DOM concentration and composition can influence microbial activity and subsequent Hg methylation.^19−23^ Conversely, eutrophication can also reduce food web MeHg accumulation due to biodilution, in which the influx of nutrients and warmer waters stimulates primary production, diluting the MeHg among the dense algal cells.,^8,24,25^

With the complex seasonality of eutrophic dimictic lakes, it can be difficult to track the fate of specific sources of Hg contamination in urban lakes through food webs. Natural abundance Hg stable isotope ratios can be used as tracers to better understand contaminant sources and transformation processes that impose subtle shifts in the relative proportions of Hg isotopes by both mass dependent fractionation (MDF, denoted as δ^202^Hg) and mass independent fractionation (MIF, denoted as Δ^199^Hg and Δ^200^Hg).^26,27^ Hg isotopic values (e.g., Δ^199^Hg and δ^202^Hg) have been used to trace habitat usage,^28,29^ fish tissue MeHg sources,^30,31^ photochemical degradation,^30^ and aquatic to terrestrial MeHg transfer.^32−34^ Although identifying Hg sources using isotopic compositions of total Hg (THg) is commonly used for fish (largely MeHg), sediments and soils (both largely iHg), there is a gap in the relationship between the iHg source preserved in sediments and soils and values recorded in fish^35^ due to isotopic fractionation occurring during methylation,^36,37^ photochemical degradation,^26,38^ and sorption.^39,40^ Additionally, previous reporting of THg and MeHg isotope values within lower trophic levels has been limited^41,42^ due to analytical limitations associated with low Hg concentrations and matrix interferences in seston, a heterogeneous mixture of organic (e.g., plankton) and inorganic particles.^43^ Hence, despite the widespread application of Hg stable isotopes, there are still gaps regarding basal food web MeHg accumulation and the potential seasonality of Hg sources within lake ecosystems.

To better understand Hg cycling, methylation, and subsequent bioaccumulation in eutrophic lakes, we assessed the seasonal transformations and sources of Hg to the aquatic food webs of two urban eutrophic lakes with historically contrasting Hg inputs (Lakes Mendota and Monona in Madison, Wisconsin, USA). We examined speciated Hg concentrations and stable isotope values in the water column and seston through two consecutive seasonal stratification cycles. We hypothesized MeHg production near the sediment surface may be an initial driver of MeHg bioaccumulation that precedes early lake stratification,^44^ but that the region of MeHg production impacting bioaccumulation shifts to the metalimnion due to Hg methylation just below thermocline driven by mineralization of labile carbon.^19,23^ We further hypothesized that differences in fish tissue Hg concentrations between the two lakes resulted from contrasting mercury contamination history (i.e., legacy Hg inputs to Lake Monona) rather than differences in biogeochemical cycling and predicted we can detect this because the sediment Hg isotope values are dissimilar. Ultimately, this study provides novel understanding of Hg cycling in understudied eutrophic lakes, confirms that water column Hg methylation is a major source to the food web, and gives us insight into future applications for Hg isotopes in complex systems.

## Methods

### Site Information

Samples for this study were collected from Lake Mendota and Lake Monona at the deepest portions (z = 25.3 and 22.6 m, respectively) of the lakes (Figure S1A). Divided by the isthmus of Madison, WI, Lakes Mendota and Monona have been well characterized temporally for chemical, physical, and biological parameters through the North American Temperate Lakes Long-Term Ecological Research (NTL-LTER) program.^45^ Both systems are temperate dimictic lakes classified as eutrophic, with dense blue-green algal and cyanobacterial blooms prevalent during summer stratification.^46^ Lake Mendota’s watershed (562 km^2^ is surrounded by urban, suburban, and agricultural areas and traps nutrients and contaminants from the upper watershed.^47−49^ Sources of Hg to Mendota include tributary inputs, direct atmospheric deposition, and overland runoff.^50^ Lake Monona, downstream from Mendota in the Yahara River system, has a smaller watershed (105 km^2^) and is surrounded mostly by urban and suburban development, with Hg inputs from legacy industrial contamination, stormwater runoff, tributary inputs, and atmospheric deposition (Figure S1B).^47,50^ As a result of their interconnectivity, both lakes exhibit similarities in hydrologic (inputs and water quality), biologic, and geologic backgrounds, but the larger watershed inputs of Mendota combined with the legacy Hg inputs of Monona allow for potential contrasts of Hg sources and biological burdens.^51,52^

### Sample Collection and Processing

All samples were collected following trace-metal clean protocols. Sample collections were as follows: waters, particulates, and bulk seston (May-October 2021, 2022), sediments (2017), and fish (2016). In brief, surface water grab samples (*n* = 35, z = 0.5 m) and water column profiles (2 profiles, 18 total samples) were obtained for the study. Profile waters were collected through acid-washed Teflon line via a peristaltic pump. It is important to note that while we were able to obtain a complete profile for Mendota (z = 0–20 m) spanning the epilimnion to the hypolimnion, we were unable to do so for Monona (z = 0–9.9 m out of 21 m), only spanning the epi- to metalimnion, due sampling logistics. In Lake Monona, biogeochemical data (e.g., temperature, dissolved oxygen, pH) was measured using a YSI Exo2 multiparameter sonde (YSI Incorporated, Yellow Springs, OH). Similar measurements were obtained for Mendota surface waters from the NTL-LTER buoy (GPS coordinates: 43.09885°, – 89.40545°) adjacent to the sampling location. Directly after sampling, waters were filtered through quartz fiber filters (QFF, 0.7 μm) into Teflon bottles and acid preserved (1% *v/v* hydrochloric acid; HCl) for filter passing MeHg (FMeHg) and THg (FTHg) analysis. QFF filters were also retained and frozen for analysis of particulate-bound Hg and MeHg (PTHg, PMeHg) as well as suspended particulate matter (SPM). One sediment core was taken at the deepest water depth of both lakes utilizing a freeze corer. In addition, within Lake Monona, a second core was taken from a depositional zone near the mouth of an important tributary to the lake, Starkweather Creek, a known historical source of Hg contamination. Cores were sliced at increasingly larger intervals from top to bottom (0.5 to 2 cm) and sections were stored frozen and later freeze-dried and homogenized. Samples were analyzed for sediment THg and MeHg (STHg, SMeHg) concentrations, THg stable isotope ratios, and percent loss on ignition (LOI).

Bulk seston was collected monthly using a plankton net (63 μm) and decanted into a cod-end bucket (52 μm), poured into a 2L polyethylene terephthalate glycol (PETG) bottle, and placed on ice. In the lab, seston were size separated through acid-cleaned Nitex mesh (243 μm followed by 63 μm) between a polyvinyl chloride (PVC) filtration apparatus. Though the seston fractions were roughly denoted as “zooplankton” (>243 μm) and “phytoplankton” (63–243 μm), larger blue-green and filamentous algae were also observed in the >243 μm fraction during late stratification. Samples were stored frozen and were later freeze-dried and coarsely homogenized using a stainless-steel blender. Fish (*n* = 16) were sampled by hook and line, white muscle tissue was collected and stored frozen until lyophilization and homogenization using a ball-mill. Fish species assessed for concentrations included *Micropterus salmoides* (largemouth bass), *Lepomis macrochirus* (bluegill), *Cyprinus carpio* (common carp), *Esox lucius* (northern pike), *Ambloplites rupestris* (rock bass), *Sander vitreus* (walleye), *Aplodinotus grunniens* (freshwater drum), *Perca flavescens* (yellow perch), and *Pomoxis nigromaculatus* (black crappie).^53^ Pelagic fish were also assessed for Hg stable isotope composition as noted below. Collections were performed by the University of Wisconsin-Madison and all procedures related to fish samples followed American Fisheries Society guidelines for the use of fish in scientific research.

### Hg and MeHg Analysis

Samples were analyzed at the U.S. Geological Survey (USGS) Mercury Research Laboratory in Madison, Wisconsin following all established standard operating procedures and quality assurance and control metrics. All quality control and assurance information for Hg concentrations measurements can be found in Section SI and Table S1.

Biological MeHg concentrations (BMeHg, fish and seston) were determined by weak acid extraction (4.5 M nitric acid; HNO_3_),^54^ ethylation, gas chromatography separation, and detection via cold vapor atomic fluorescence spectroscopy (CVAFS, limit of detection 1.3 ng g^–1^). After successfully measuring BMeHg, biological extracts were oxidized with sodium persulfate (2 M, 5% *v/v*) and bromine monochloride (BrCl, 10% *v/v*) to degrade organic carbon prior to biological THg (BTHg) analysis. Oxidized sample extracts were analyzed via tin reduction coupled to gold amalgamation prior to quantification via CVAFS using U.S. Environmental Protection Agency (EPA) Method 1631.^55^ FMeHg, PMeHg, and SMeHg samples were prepared for analysis using a modified version of EPA Method 1630, which uses isotope dilution coupled to quantification via inductively coupled plasma mass spectrometry (ICP-MS).^56^ Water samples for FTHg and PTHg were oxidized with BrCl (1%, and 5% *v/v*, respectively) and analyzed following U.S. EPA Method 1631. STHg was analyzed with direct combustion paired with cold vapor atomic absorbance spectroscopy.^57^ Dissolved organic carbon (DOC) was measured using standard U.S. EPA Method 415.3.^58^ LOI of sediments was determined as the mass lost following heating of a dried sample at 550°C for 2 h.

### Hg and MeHg Stable Isotope Analysis

For THg isotope ratio measurements, seston were microwave digested in 5 mL reverse aqua regia (3:1 HNO_3_ to HCl), diluted, filtered, and oxidized with BrCl (5% *v/v*) according to Section SII and Table S2. Fish and sediments were digested following acid digestion protocols outlined elsewhere protocol (Section SII).^59^ A subset of seston samples were prepared for MeHg stable isotopic analyses using a modified distillation method (EPA Method 1630, Section SIII).^43,60^ Due to sample mass limitations and low BMeHg concentrations of 63–243 μm seston samples, mostly >243 μm samples (*n* = 4) were chosen for MeHg stable isotope analysis (in addition to one 63–243 μm sample). All sediment and seston digests too low for direct analysis (<15 ng of Hg per digest) were preconcentrated using a purge and trap method to reach the analytical threshold for multicollector ICP-MS (MC-ICP-MS) detection.^61^ National Institute of Standard and Technology (NIST) 3133 and internal reference materials (MSC192AZ, seston >243 μm) were also included to ensure that no isotopic fractionation was induced by preconcentration methods (isotopic values and recoveries listed in Table S3).^61^

THg and MeHg stable isotope ratios were measured using a MC-ICP-MS (Neptune Plus, Thermo Scientific) equipped with a custom gas liquid separator.^62^ To correct for any mass bias, thallium (40 ng mL^–1^, NIST 997) was introduced into the gas liquid separator in tandem with Hg introduction. All samples were matrix-matched (∼5% *v/v* acid content) and concentration-matched (0.5 to 1 ng mL^–1^) to the NIST 3133 bracketing standard. Operating parameters and cup configurations for the MC-ICP-MS used previously established methods.^36,62^ Every five samples, a secondary standard (NIST RM 8610, “UM-Almaden”) was measured to evaluate instrument precision and accuracy (δ^202^THg = – 0.54 ± 0.08‰, Δ^199^THg = – 0.01 ± 0.05‰, Δ^200^THg = 0.01 ± 0.05‰, Δ^201^THg = – 0.04 ± 0.06‰, and Δ^204^THg = – 0.02 ± 0.10 ‰; 2 standard deviation (SD), *n* = 90), values were consistent with previous publications.^63^ All Hg stable isotope data is reported using previously established conventions noted in Section SIV.^64^ Certified reference materials were analyzed with each sample batch to assess processing efficiency, MeHg and THg isotope values for biota and sediments (Table S3) were similar to previous literature values.,^35,56,63^ All Hg species concentrations, Hg stable isotope ratios, and QA/QC data are available in the associated Supporting Information or data release.^65^

### Data Processing

For each sampling event, the following calculations were (see Section SV for more information): (1) partitioning coefficient (*K*_d_) for MeHg transfer from aqueous to particulate (0.7 μm), (2) bioaccumulation factor (BAF) seston uptake of MeHg from water to the 63–243 μm seston fraction, and (3) biomagnification factor (BMF) for MeHg transfer from the 63–243 μm to the 243 μm seston fractions (Tables S4 and S5).^7^ Secchi depth (a measure of water clarity), water temperature and dissolved oxygen profiles corresponding with sampling dates were also plotted for each lake (Figures S3–S5), the data obtained from the NTL-LTER database.^66,67^

The relative source contribution (epilimnetic versus benthic) of MeHg to the pelagic fish from both lakes was calculated using Δ^199^Hg (encompassing THg and MeHg, as specified below) values using a binary mass balance approach (eq 1):
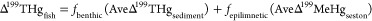
1

Where *f*_*epilimnetic*_ and *f*_*benthic*_ are the fraction of epilimnetic and benthic-sourced MeHg for each fish. Δ^199^THg_fish_ is the measured value for the specific fish, whereas the AveΔ^199^THg_sediment_ (surface sediment, z = 1–5 cm) and AveΔ^199^ MeHg_seston_ are lake-specific averages for each matrix. We used Δ^199^Hg instead of δ^202^Hg because water column photochemical differences were easier to distinguish between the two matrices (e.g., seston and sediments) than source and process-based isotopic fractionation associated with δ^202^Hg.e.g.,^36,40,68,69^ In this approach, we assume that Δ^199^THg_fish_ ≈ Δ^199^ MeHg_fish_ because fish (top predator species) are typically >95% MeHg.^70^ Similarly, we assume Δ^199^THg_sediment_ ≈ Δ^199^ MeHg_sediment_ because we do not expect MeHg in sediment to undergo additional mass independent fractionation (MIF, Δ^199^Hg) during microbial methylation or be subject to MIF due to photochemical loss.^36,37^ For this calculation, we used pelagic fish species including largemouth bass, bluegill, northern pike, rock bass, walleye, freshwater drum, yellow perch and black crappie (Table S6). Notably, benthic fish species (carp) were omitted from this calculation, as the inputs are not representative of the carp diet (e.g., detritus).

## Results and Discussion

### Sediment Source History of the Lakes

Sediments from Lakes Mendota and Monona reflect historical contrasts in Hg inputs and sources therein (Figure S2). Elevated STHg concentrations in Lake Monona sediments can be observed from depths of 85–58 cm, peaking at 1,800 ng g^–1^ at 58 cm (Figure S2A), corresponding to historical industrial inputs entering the lake. From 58 to 30 cm, we observe a decline in the STHg concentrations in Lake Monona sediments, potentially indicating a decline in Hg effluent from industrial sources. In contrast, Lake Mendota sediment THg concentrations have remained consistent (119.6 ± 64.0 ng g^–1^) through time, peaking at 180.7 ng g^–1^ (49 cm), ∼ 10x lower than the highest STHg concentrations observed in Monona. In more recently deposited surface sediments (1–5 cm), significantly lower (*p* < 0.05) STHg concentrations were measured in Lake Mendota (53.9 ± 193 ng g^–1^, 1SD, *n* = 9) compared to Monona (176.3 ± 62.9 ng g^–1^ at deep hole, 417.4 ± 29.2 ng g^–1^ near Starkweather Creek; 1SD, *n* = 3 and 5, respectively), despite similar organic carbon content in both lakes’ sediments (average 19.5 ± 1.4% LOI for both lakes). The THg isotopic composition of δ^202^THg in surface sediments (−1.05‰ Mendota, – 0.45‰ Monona, z = 2 cm; Figure S2B) suggests recent sedimentary sources are different between the lakes; specifically, sources were watershed^71,72^ and industrial-derived,^59,73,74^ in Mendota and Monona, respectively. The different sources inferred from the isotopic ratios allow us to examine the bioavailability of ongoing releases of legacy Hg from sediment resuspension and Hg derived from contemporary overland runoff and direct atmospheric sources. More detailed information regarding THg concentrations and isotope values at depth for the Lakes Mendota and Monona sediment cores can be found in Section SVI.

### Assessment of Seasonal THg and MeHg Concentrations in Lakes Mendota and Monona

#### Waters and Particles

**Figure 1:**
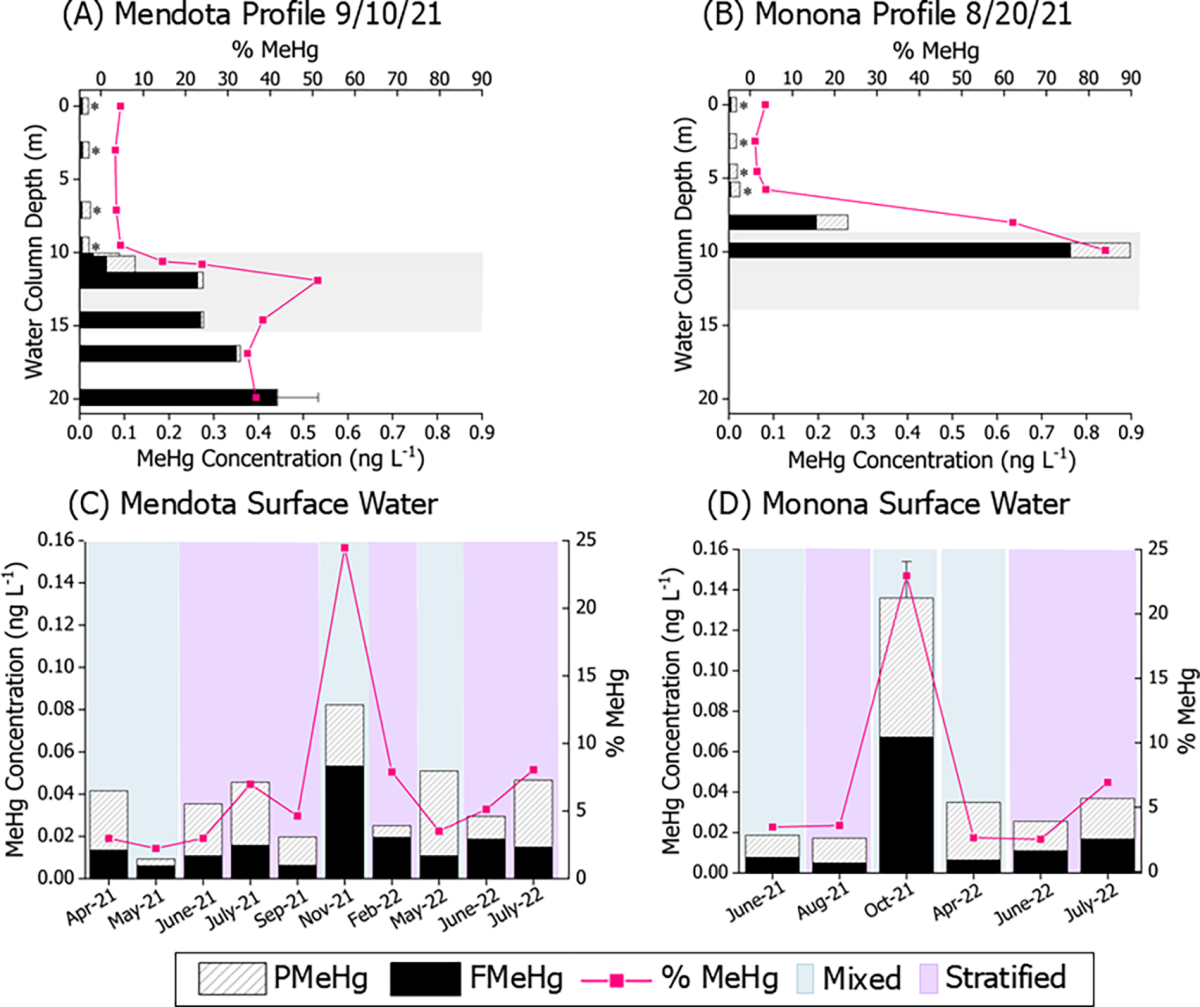
Methylmercury concentrations in profile and surface waters. Profile waters were obtained from (A) Mendota in early September 2021 and (B) Monona in late August 2021. Shaded regions in panels (A) and (B) represent the metalimnia of the lakes on the sampling date (Figures S 3f, and S4e). Surface waters were collected monthly from 2021 to 2022 from (C) Lake Mendota and (D) Lake Monona. Colored bars in plots (C) and (D) represent lake water conditions: mixed (blue) and stratified (purple) as determined from the LTER water temperature and dissolved oxygen profiles (Figures S3 and S4).^66^ Stacked bars reflect mercury speciation of particulate methylmercury (PMeHg) and filtered MeHg (FMeHg) in the waters, while the pink line reflects percent MeHg (%MeHg). Error bars in panels (A) and (D) represent 1 SD PMeHg + 1 SD FMeHg of water replicates (*n* = 2 for each 1SD error bar).

Seasonal surface waters reveal distinct physical and biogeochemical processes illustrative of a dimictic eutrophic lake (Figures S3 and S4). Notably, both lakes fully mix in spring (∼April) and fall (∼October/November) (Figures S3b,g and S4b,g) and from midsummer to early fall, both strongly stratify thermally (e.g., Figures S3e,f and S4e,f). Hg speciation profiles during stratification show the highest FMeHg concentrations in the meta- and hypolimnia of both lakes, peaking at 0.44 ± 0.09 ng L^–1^ (Mendota, z = 19.9 m, 1SD, *n* = 2), and 0.76 ng L^–1^ (Monona z = 9.9 m). (Figure 1A,B). Previous work has shown that FMeHg concentrations are highest during this late stratification period.^23^ We also noted peak %MeHg in the metalimnia of both lakes, 51% in Mendota (z = 11.9 m) and 84% in Monona (z = 9.9 m) during stratification. These measurements were consistent with previously identified zones of water column methylation in Lake Mendota.^19,23^ In general, buildup of MeHg and THg in lower meta- and hypolimnion is often ascribed to Hg diffusion from surface sediments^75^ but could also be derived from water column methylation, due to mineralization of settling particulate matter, including plankton, which remobilizes iHg and drives the anaerobic conditions and metabolism that are linked to methylation.^23,76−78^ The peak in %MeHg in the metalimnia of both lakes also supports that mineralization and remobilization of iHg bound to fresh and decaying organic matter (e.g., plankton) at density gradients is also an important source of MeHg production.^19,23,76,79^

Suspended particulate matter-water partitioning coefficients aid in potentially diagnosing whether mineralization drives the phase-distribution of iHg and MeHg in the water column.^80−82^ MeHg partitioning from aqueous phase to particulate phase, as measured by the coefficient log*K*_d_, was on average 5.40 ± 0.63 for surface waters (z = 0.5 m; Table S4), similar to values from Great Lakes surface waters, ranging from 5.4 to 6.0.^7,80^ Log*K*_d_ also decreased with depth, as observed in Mendota (log*K*_d_ = 5.57 at z = 0 m, log*K*_d_ = 3.71 ± 0.04 at z = 19.9 m) and Monona (log*K*_d_ = 5.81 ± 0.78 at z = 0 m, log*K*_d_ = 4.98 at z = 19.9 m) (Table S5), indicating that MeHg in these systems partitions to particulate matter in the upper waters more strongly than in deeper waters, further emphasizing the importance of mineralization and remobilization of MeHg in the meta- and hypolimnion. Relatively higher peak FMeHg and PMeHg (0.76 and 0.13 ng L^–1^, respectively) were observed in the metalimnion of Lake Monona compared to Lake Mendota’s peak concentrations in the meta- and hypolimnion (0.51 and 0.06 ng L^–1^). While the exact mechanism driving this difference is unknown, changes in the relative amount of FMeHg and PMeHg could be due to differences in the urban runoff component or differences in settling efficiencies of particles between the two lakes.^83^

Surface (z = 0.5 m) waters of both lakes were similar for FTHg (0.27 ± 0.09 ng L^–1^, *n* = 18) and for FMeHg (0.02 ± 0.02 ng L^–1^, *n* = 18) (Figure 1C,D), which are comparable to previous measurements of inland Minnesota lakes (FTHg = 0.22 ± 0.11 ng L^–1^, *n* = 31; FMeHg = 0.01 ± 0.02 ng L^–1^, *n* = 30).^84^ We observed heightened FMeHg in surface waters (0.05 ng L^–1^ in Mendota, 0.07 ± 0.02 ng L^–1^ in Monona; 1SD, *n* = 2) during the period following fall turnover (Figure 1C,D). We hypothesize that the concentration maxima during mixing results from the release of MeHg-rich meta- and hypolimnetic waters (Figure 1A,B).^23,81^ The highest %MeHg values in surface waters (average 23.9 ± 6.5%, 1SD, *n* = 3) also occur during fall turnover in October and November for both lakes.

#### Food Web

**Figure 2:**
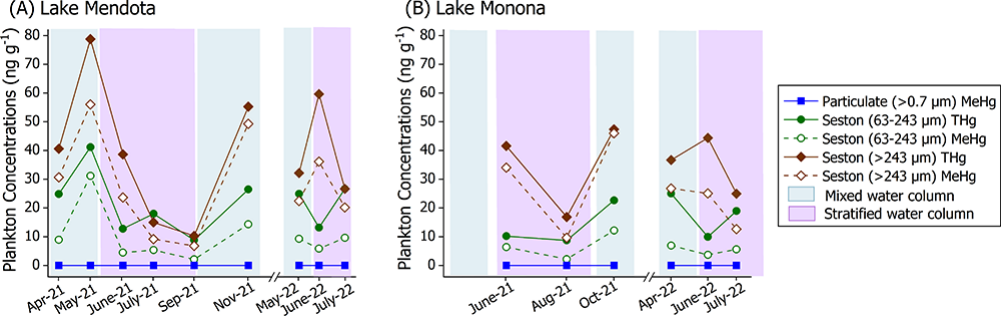
Total mercury (THg) and methylmercury (MeHg) concentrations in seston fractions. Fractions of seston (63–243 μm ∼ “phytoplankton”, > 243 μm ∼ “zooplankton”) were sampled monthly from (A) Lake Mendota and (B) Lake Monona in 2021, and 2022. Colored bars represent lake water conditions: mixed (blue) and stratified (purple) as determined from the LTER water temperature and dissolved oxygen profiles (Figures S3 and S4).^66^

MeHg burdens in seston in Lakes Mendota and Monona vary seasonally, likely due to fluctuations in algal community composition and density, lake mixing dynamics, and the MeHg supply. In general, we observed that concentrations of BTHg and BMeHg in seston were comparable in the two lakes despite higher aqueous and sediment concentrations in Lake Monona. Consistent with biomagnification, we observed higher BTHg and BMeHg concentrations in >243 μm “zooplankton” (BTHg 10.3–78.8 ng g^–1^, BMeHg 6.8–56.0 ng g^–1^, %MeHg 53.1–89.1%) compared to 63 to 243 μm “phytoplankton” (BTHg 8.6–41.2 ng g^–1^, BMeHg 2.17–31.2 ng g^–1^, %MeHg 23.7–75.7%) (Figure 2). Seston concentrations in this study were also in the range of previous zooplankton measurements from inland Wisconsin lakes (12–206 ng g^–1^ BTHg, 5–161 ng g^–1^ BMeHg),^5^ the basins of Lake Champlain (18.8–68.1 ng g^–1^ BTHg, 1–47 ng g^–1^ BMeHg),^8^ and other lake studies.^81,85^

Seston BTHg and BMeHg concentrations peak during fall turnover in both lakes, a critical period for uptake and biomagnification,^10^ with zooplankton having the highest %BMeHg (89.1% and 97.2%, Mendota and Monona respectively) during this period, also corresponding with observed elevated FMeHg in epilimnetic waters (Figure 1C,D). Similarly, seston BTHg and BMeHg concentrations are also elevated during spring mixing, when phytoplankton have the highest %BMeHg (Mendota 75.7% and Monona 62.5%), indicating that MeHg production or release during ice cover is important for bioaccumulation in spring (Figure 2). In contrast, lower MeHg fractions were evident during midsummer stratification (July) in both lakes, with zooplankton %BMeHg averaging 60.2 ± 1.2% and 53.1% and phytoplankton averaging 34.0 ± 6.2% and 32.0% in Mendota (*n* = 2) and Monona, respectively. We hypothesize that the decrease in MeHg supply in the epilimnion likely stems from loss mechanisms like photodegradation, which is especially prominent in surface waters.^42,86−89^ Additionally, there may also be a decrease in diffusion of the metalimnetic/benthic-sourced MeHg during stratification. Instead, epilimnetic MeHg supply results from methylation in oxic to suboxic metalimnetic waters of iHg delivered from the watershed and from atmospheric deposition that can partition to particles, settle, and be added to the pool of Hg that can be methylated in the water column.^7,76^

Biodilution in the epilimnia of these two eutrophic systems can also play an important role in bioaccumulation during stratification. Dense plankton blooms (mostly blue-greens, filamentous algae, cyanobacteria)^90,91^ reduce BMeHg burdens due to biodilution, demonstrated elsewhere by negative correlations between Hg concentrations and algal biomass.^8,92^ Biodilution of MeHg can be observed via the MeHg logBAFs (aqueous → 63–243 μm “phytoplankton”; Table S4), which were similar to previously studied Wisconsin lakes (logBAF = 4.8–6.2),^5^ and were highest during early spring mixing in April/May (6.15 ± 0.48, Mendota, 1SD, *n* = 3; 6.03 Monona, *n* = 1) and lower through the rest of the season June-November (5.55 ± 0.11, Mendota, 1SD, *n* = 6; 5.57 ± 0.24, Monona, 1SD, *n* = 5). The higher spring logBAF values also coincide with lower SPM values (2.00 ± 1.09 mg L^–1^, Mendota, 1SD, *n* = 3; 3.93 mg L^–1^ Monona; Table S4), suggesting that the elevated logBAF values may be due to, in part, enhanced grazing of plankton on the particles at this time and consequentially, less competition between phytoplanktonic uptake of MeHg and sorption of MeHg to particles. Since DOC remained relatively constant (4.77 ± 0.40 mg L^–1^, 1SD, *n* = 18) across the seasons, we can attribute the difference between spring and summer/fall MeHg logBAFs to biodilution rather than DOC dependence.^5,7^ Further, MeHg logBAFs from June-November were similar to particulate log*K*_*d*_ values of 5.55 ± 0.11 and 5.63 ± 0.43 (Mendota and Monona, respectively, Table S4). Conversely, MeHg logBMFs (63–243 μm → > 243 μm plankton) were variable throughout the year, ranging from 1.72 to 5.31. The variance in these values can be explained by the seasonal shifts in trophic complexity captured by the >243 μm fraction, with the taxonomic diversity spanning organisms across various consumer trophic levels (e.g., daphnia, blue green algae, spiny waterflea).^90,91^ This further emphasizes that plankton community composition highly influences our interpretation of the biomagnification rates of MeHg into upper trophic levels.^7^

To further evaluate MeHg bioaccumulation in Lakes Mendota and Monona, we measured the THg content of fish (Table S6). In Lake Mendota, fish BTHg ranged from 700.0 to 3,246 ng g^–1^ (average 1,455 ± 914.6 ng g^–1^; 1SD, *n* = 7) whereas Monona fish ranged from 253.9 to 1,320 ng g^–1^ (average 546.8 ± 355.0 g^–1^; 1SD, *n* = 9). Notably, fish collected from the two lakes were samples of opportunity and spanned a wide range of sizes, feeding guilds, and species (e.g., bluegill vs carp vs walleye). In general, as pelagic fish length enlarged, BTHg also increased (Figure S5), consistent with previous studies.^93,94^ However, we could not determine a length-BTHg relationship, as multiple of each species would be required for a proper statistical assessment. It is likely that the elevated concentrations (and larger range) observed in Lake Mendota fish is due the higher quantity of larger sized fish and variety of species analyzed (Table S6).

### Using Hg Stable Isotopes to Assess Sources and Processes Impacting MeHg Bioaccumulation

**Figure 3:**
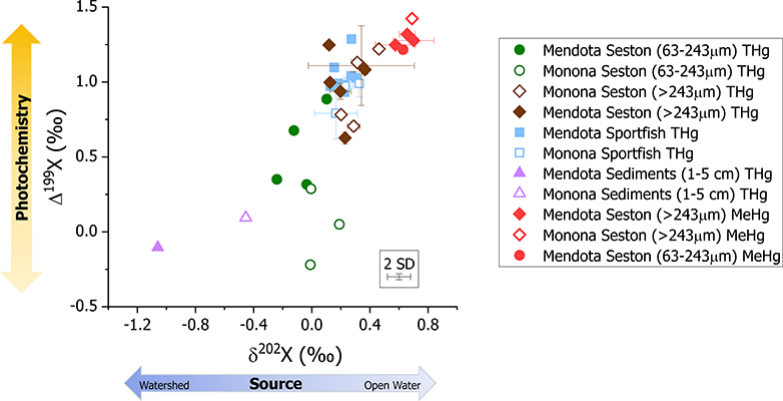
THg and MeHg stable isotope plots for sediment, seston, and fish collected from Lakes Mendota and Monona. (A) biplot of Δ^199^X vs δ^202^X isotopic composition, where X is mercury species: THg or MeHg, of sediments, seston (63–243 μm ∼ “phytoplankton”, > 243 μm ∼ “zooplankton”), and fish from both lakes. Error box represents the 2SD of certified reference material IAEA SL-1 (which had the largest SD of all of the matrix CRM’s) measurements of isotopic ratios of Δ^199^THg and δ^202^THg and error bars represent ± 1 SD of sample replicates or composites. Isotopic data can be found in associated data releases.^53,65^

The seasonal Hg species patterns in seston allowed for the examination of temporal dynamics in Hg isotope values that have not been previously explored. While δ^202^Hg is typically used to examine environmental sources and processing, we determined that the breadth of ongoing processes (e.g., methylation, sorption, reduction, etc.) would be difficult to ascertain at a seasonal scale. Thus, we aimed to examine seasonal variation attributed to photochemical demethylation in the water column, which can be assessed using Δ^199^Hg (Figure S6). Furthermore, Δ^199^Hg values remain relatively constant in biota despite processes that induce MDF occurring in the food web.^26^ When examining THg and MeHg isotope values in seston (>243 μm), the average Δ^199^ MeHg and Δ^199^THg value (0.97 ± 0.21‰, *n* = 10) was similar to the Δ^199^THg values in fish tissues (0.96 ± 0.12‰, *n* = 16) (Figure 3). Additionally, the photochemical slope (Δ^199^Hg/ Δ^201^Hg) including both THg and MeHg isotope values for all samples was 1.30 ± 0.07 (*n* = 34), which is indicative of photochemical demethylation, aligning with previous laboratory investigations (Figure S7).^26,38^

At lower trophic levels, we observed seasonal differences in Δ^199^THg values between phytoplankton and zooplankton size fractions in both lakes (Figure S6), possibly due to changes in %BMeHg content of the phyto- and zooplankton as well as solar irradiance, as affected by water clarity. We predicted that Δ^199^THg values in seston would fluctuate as a function of time, due to changes in seasonal light dynamics linked to productivity or solar radiance (Figure S8) as well as possible MeHg production in benthic/hypolimnetic (assumed to be near zero Δ^199^THg) versus meta/epipelagic (assumed to be more positive Δ^199^THg) zones. Some of the highest Δ^199^THg values (1.11 ± 0.12‰ zooplankton, 0.83 ± 0.14‰ phytoplankton) (Figure S6), coincide with “clearwater phase” as demonstrated by the increase in Secchi depth, or water clarity, in late April/early May (Figure S8). The spring clearwater phase is characterized by low biomass of phytoplankton, induced by zooplankton (e.g., copepods and Daphnia) grazing on phytoplankton and consequentially, high water clarity and light penetration into the water column.^16−18^ We surmise that higher seston Δ^199^THg values observed at this time are, in part, due to greater penetration of sunlight into the water column (increased water clarity), which leads to enhanced photodegradation, driving odd-MIF.^26^ The lowest Δ^199^THg values in both size fractions were observed during late summer/early fall, a period of dense blooms and filamentous algae, which shade MeHg from demethylation and reduce odd-MIF, an observation confirmed by previous laboratory studies.^42^ The decrease in Δ^199^THg values during fall in Mendota, especially in phytoplankton (Figure S6B), could also result from turnover exposing plankton to hypolimnetic MeHg that has not been photochemically processed to the same extent as MeHg in epilimnetic zones.

Differences in Δ^199^THg values between phytoplankton and zooplankton where also observed within a sampling event, which is likely due differences in %BMeHg between zooplankton than phytoplankton. While trophic transfer does not induce fractionation of Δ^199^ MeHg (values are conserved during trophic magnification of MeHg between predators and prey),^95,96^ we cannot definitively show this in seston because the THg in these organisms is not entirely MeHg. Specifically, phytoplankton and zooplankton typically have mixed iHg and MeHg contributions that could have differing Δ^199^Hg values, complicating direct comparisons between the two groups.^56^ This difference possibly arises because the bioaccumulated MeHg pool is exposed to different processes that induce fractionation (e.g., photochemical demethylation) compared to the iHg (e.g., photoreduction) pool, resulting in differences between zooplankton that have higher MeHg content and phytoplankton which have lower MeHg content. However, these subtle differences cannot be resolved by examining photochemical slopes of our samples (Figure S7).^26^ Further, iHg may be associated with smaller inorganic particles that are more difficult to separate from phytoplankton cells during seston collection, as these inorganic particles may also have lower Δ^199^THg values.^97^ To further assess isotope differences between iHg and MeHg pools within in an organism, we performed direct isotope measurements on isolated MeHg from a subset of zooplankton and phytoplankton samples as mass allowed.,^43,56,98^ We observed that the measured MeHg stable isotope ratios in these lakes have consistently higher Δ^199^ MeHg values compared to the Δ^199^THg values (Figure S9), also noted previously.^56^ Pairing this observation, along with the dissimilar %BMeHg observed between phytoplankton and zooplankton (a 34.1 ± 8.9% difference), we posit that the elevated Δ^199^THg in zooplankton is likely due to the higher %MeHg, because MeHg undergoes heightened photochemical fractionation. In other words, as the MeHg is bioaccumulated, the more-photochemically processed Δ^199^ MeHg values raise the overall Δ^199^THg signal. Ultimately, with the variable %BMeHg, MeHg supply, biodilution, and water clarity considered, it is clear that seasonal zooplankton and phytoplankton display differing isotopic composition, an important factor to consider when utilizing Hg stable isotopes for food web MeHg source attribution in the lower food web.

To further deconvolute Hg sources to the food web within both lakes, we compared Δ^199^Hg and δ^202^Hg isotope values across matrices including sediments, seston, and fish (Figure 3). As mentioned previously, isotope values of Lake Mendota and Monona surface sediments show contrasting δ^202^THg values, with Lake Monona δ^202^THg (−0.45 ± 0.01‰) skewing more toward values observed in industrially contaminated sites whereas Lake Mendota (−1.05‰) is more comparable to previously measured background sites experiencing large sediment contributions from the watershed without point source contamination (Section SVIII and Figure S2B).^73^ Furthermore, the sediment exhibited only minimal near-zero Δ^199^THg values (ranging – 0.10 to 0.19‰). Despite the contrasting Hg sources in recent sediments of the lakes, the food web Δ^199^THg and δ^202^THg values are similar for each biota type, as indicated by the overlapping isotope values in phytoplankton, zooplankton, and fish for both lakes (Figure 3). We surmise that overlapping Hg isotope values across biota from both lakes suggests the bioaccumulation of a similar Hg source. This also suggests that legacy contaminated sediments in Lake Monona are not substantially contributing to observed seston MeHg, but rather during stratification, atmospheric Hg is the main source of bioaccumulative MeHg to the food webs, as further supported by slightly positive (>0.05‰) Δ^200^THg values (which trace atmospheric transformations occurring during Hg transport)^71,99^ in seston from both lakes (Figure S10).

To gain more insight on epilimnetic versus benthic Hg sources across the food webs, we applied a mass balance approach using Δ^199^Hg to determine the proportion of MeHg in fish attributable to epipelagic (atmospheric, mineralized seston) and benthic (sediment) sources (eq 1). Using similar pelagic fish from each lake, we can apportion the MeHg source (pelagic vs benthic-derived) for each fish in both lakes. We calculated that Lake Mendota fish contained an estimated average of 81 ± 4% pelagic and 19 ± 4% benthic-derived MeHg (1SD, *n* = 7) whereas Monona fish contained 67 ± 5% pelagic and 33 ± 5% benthic MeHg (1SD, *n* = 6) (Table S6). These results agree with the above conclusion that sediments are not the dominant contributor to food web Hg within both these lakes. The higher proportion of benthic MeHg within the Lake Monona food web may suggest that legacy Hg contamination within sediments may continue to contribute a small amount to contemporary fish concentrations. The larger proportion of benthic MeHg food web contribution in Lake Monona may also explain the previous existence of more-stringent fish consumption advisories on the lake compared to the general advisory that includes Lake Mendota.^52,100^ However, we do note that other ecological factors between the lakes may also contribute to differences between fish Hg content.

## Conclusions

Ultimately, this study illustrates how the seasonal complexity of eutrophic dimictic lakes impacts Hg cycling, food web burdens, and source reactivity. By utilizing lower trophic level biota (seston), we can gain a finer temporal understanding of Hg dynamics when compared to fish, which integrate Hg sources over a longer period of time. It was observed that MeHg produced in the water column, driven by mineralization, is an important source of MeHg to plankton and eventually, fish within eutrophic dimitic lakes, as confirmed by the Δ^199^Hg source apportionment model. By leveraging Hg species concentrations and stable isotope values, this work also highlights that spring overturn releases a key pulse of MeHg that readily enters the food web and that fall mixing as a critical time of MeHg dispersion into the water column and lower food web.^10,19,23,81^ Lastly, we also demonstrate that water column cycling within these lakes promotes the bioaccumulation of atmospheric Hg sources and closely connects the food web to pelagic Hg cycling, especially during summer stratification when productivity is greatest. Spring plankton blooms have been occurring earlier due to climate change; with ice-off arriving sooner, phytoplankton thrive with exposure to light, warmer temperatures, and available nutrients (e.g., phosphorus, nitrate, silicon).^101,102^ Additionally, interannual changes in lake thermal structure has been shown to drive fluctuations in summer anoxia in eutrophic systems, potentially influencing Hg methylation.^103^ These components add further complexity to eutrophic systems and our data suggests that there is a need for future winter limnological investigations to understand Hg cycling prior to spring planktonic uptake. With the continued combined approach of concentration and isotope data, we can better elucidate cycling and bioaccumulation pathways of MeHg in dynamic lake ecosystems, key for the future management of Hg contamination, especially in complex systems like eutrophic lakes.

## Supporting Information Available

Additional sampling, sample processing and analysis methods, tables, and figures (PDF)
