# Supplementary material for: Seasonal Stratification Drives Bioaccumulation of Pelagic Mercury Sources in Eutrophic Lakes
Source: ACS ES T Water. 2025 Apr 10;5(5):2444–54. doi: 10.1021/acsestwater.5c00028 (PMC12070409; doi:10.1021/acsestwater.5c00028)
Supplement: Supplementary file 1 — ew5c00028_si_001.pdf [file ew5c00028_si_001.pdf]

Supporting Information for "Seasonal Stratification Drives Bioaccumulation of Pelagic Mercury Sources in Eutrophic Lakes"

Grace J. Armstrong[1,2*], Sarah E. Janssen[1], Ryan F. Lepak[3], Tylor J. Rosera[1], Benjamin D. Peterson[4,5], Samia T. Cushing[6], Michael T. Tate[1], James P. Hurley[2,7]

[1]*U.S. Geological Survey, Upper Midwest Water Science Center, Madison, WI, 53726, USA*
[2]*Environmental Chemistry and Technology Program, University of Wisconsin-Madison, Madison, WI, 53706, USA*
[3]*U.S. Environmental Protection Agency, Duluth, MN, 55804, USA*
[4]*Department of Bacteriology, University of Wisconsin–Madison, Madison, WI, 53706, USA*
[5]*Department of Environmental Toxicology, University of California-Davis, Davis, CA, 95616 USA*
[6]*Freshwater@UW Summer Research Opportunity Program, University of Wisconsin-Madison, Madison, WI, 53706, USA*
[7]*University of Wisconsin Aquatic Sciences Center, Madison, WI, 53706, USA*
[*]*Corresponding author*

Contains: Supporting methods, **6** supporting tables, **10** supporting figures, **21** pages

Any use of trade, firm, or product names is for descriptive purposes only and does not imply endorsement by the U.S. Government. All data can be found in the corresponding U.S. Geological Survey data releases.[1,13]

SUPPORTING METHODS

Section I. Quality Control and Assurance for Mercury (Hg) Concentration Analyses

The USGS Mercury Research Laboratory (MRL) conducts analysis on both naturally abundant and isotopically enriched samples. To avoid any possible isotopic contamination, both analytical routes are conducted in separate lab spaces using different reagents and equipment allotted for enriched and non-enriched samples.[2]

Quality assurance and quality control (QA/QC) metrics are typically monitored throughout the course of analysis by replicates, spikes, methods blanks, calibration blanks, certified reference materials (CRMs), but varies depending on the analysis. When suitable CRMs cannot be obtained due to the nature of the matrix (e.g., different tissue type) or Hg concentrations (e.g., substantially lower, or higher Hg concentration than the samples) then in-house internal standards are used. For these analyses the following CRMs were used: in-house reference “MSC192AZ-Mendota Zooplankton” (THg = 70.3±7.8 ng g^{-1}, MeHg = 49.3±3.5 ng g^{-1}) and International Atomic Energy Agency (IAEA)-452 for seston samples; IAEA-407 for fish, and IAEA SL-1 for sediments. All CRM analyses were in agreement with the certified values (within 80-120% recovery) or previously established concentrations, in the case of the in-house standard (**Table S1**).

In addition, all analyses conducted by USGS MRL adhere to strict quality assurance and controls including calibration blanks, method blanks, standard calibration ($r^2 > 0.995$ for linear standard curves), quality control standard (e.g., secondary standard) recovery (90-110%), and sample replication measurements (<10% difference). Quality control checks are performed every 5-10 samples depending on analyses.

Section II. THg Isotope Sample Digestion

Seston samples (>243 and 63-243μm fractions) were prepared for Hg isotopic analysis via microwave digestion. Seston has previously been a difficult matrix to prepare for isotopic analysis due to the low Hg concentrations, high organic carbon content, additional chemical components (e.g., silica) that add to matrix complexity, as well as high mass requirement to meet the detection threshold of the multi-collector inductively coupled plasma mass spectrometer (MC-ICP-MS). Hence, microwave digestion was employed to ensure that the sample was effectively digested. Depending on sample matrix, a variety of different acids or oxidants (e.g., peroxide) can be utilized for microwave digestion. We tested commonly used digestion approaches to determine which had the most consistent recovery for seston matrices. Based on microwave digestion recovery, success of downstream preconcentration steps, and visual components reverse aqua regia (3:1 nitric acid [HNO_3] to hydrochloric acid [HCl]) was selected as the optimal acid combination for digesting seston (**Table S2**). For seston, ~300-500 mg of sample was weighed into each Teflon microwave vessel and 5 mL of reverse aqua regia was added. Samples were loosely capped in a fume hood and left to sit for about an hour to allow for off-gassing to reduce pressure hazard and potential sample loss while opening the vessels after the digestion. After an hour, microwave vessels were sealed and samples were digested at 210ºC

(1800 W, 20-minute temperature ramp-up followed by 15-minute hold at 210ºC). After the cool-down period, samples were carefully opened and decanted into a 20 mL glass vial and diluted with 4 mL reagent water. Samples were then filtered and brominated (5% BrCl).

Fish tissues were prepared by hot acid digestion following previously published protocols.[3] Specifically, fish tissue samples were digested in concentrated HNO_3 at 90ºC for 8-10 hours while loosely capped. Extracts were then fully oxidized with bromine monochloride (BrCl) to 10% of the total volume and heated on the block for an additional 2 hours followed by 50% dilution with reagent water. Sediment samples were digested in 5 mL of aqua regia (3:1 $HCl:HNO_3$) at 90ºC for 12 hours while loosely capped to avoid back pressure. After cooling, samples were diluted with reagent water to 50% acid content.

After sample extraction, via microwave or hot acid digestion, sample extracts were analyzed for total mercury (THg) concentration before proceeding to MC-ICP-MS analysis (fish and sediments) or sample preconcentration (seston).[4]

Section III. Methylmercury (MeHg) Isotope Preparation of Seston by Distillation

Seston MeHg stable isotope samples were prepared using a modified distillation method.[5, 6] Due to mass limitations, the majority of MeHg stable isotopic analysis was conducted on the >243μm size fraction with a few 63μm samples selected as a trophic comparison. Briefly, samples were weighed out into 125 mL distillation vials, aiming for ~10-15 ng of MeHg total per sample. Because of this, some samples were spread across multiple distillation vials so as not to exceed about 100 mg per distillation vial. Additional CRMs (IAEA-452), replicates, and method blanks were included in the distillation setup for QA/QC. The in-house standard was also included to assess consistency between runs within a seston matrix. CRM recoveries for THg and MeHg stable isotopes are denoted in **Table S3**. Distillation procedures follows those outlined in Rosera et al. 2020,[6] which are a modification of Environmental Protection Agency Method 1630. Samples were assessed for MeHg and THg concentrations after distillation to MeHg check to evaluate recoveries and potential remaining inorganic Hg. Because the inorganic recoveries roughly equaled the MeHg recoveries, it was determined that no additional processing was required.[6]

For QA/QC, ambient MeHg isotope recoveries adhered to 85-115% whereas CRMs had 90-110% recoveries. Ambient sample recovery requirements have a larger range of passing criteria due to the difficulties of homogenizing a representative sample (e.g., slight variations between MeHg concentrations between each sample weigh-out), as detailed elsewhere.[2]

Section IV. Reporting Conventions for Hg Stable Isotopes

All Hg stable isotope data is reported using previously established conventions.[7] Mass dependent fractionation (MDF) was calculated as:

$$\delta^{XXX}Hg\ (‰) = [(^{XXX}Hg/^{198}Hg_{sample})/(^{XXX}Hg/^{198}Hg_{NIST3133})-1]*1000 \qquad \textbf{(Eq. S1)}$$

where the isotope of interest is represented by XXX, whereas Hg mass independent fractionation (MIF) was calculated by:

$$\Delta^{XXX}Hg(‰) = \delta^{XXX}Hg - (\delta^{202}Hg*\beta) \qquad \textbf{(Eq. S2)}$$

where β is the mass scaling factor, calculated as explained by Blum et. al 2007.[7] The precision and accuracy of isotope measurements was assessed using National Institute of Standards and Technology (NIST) RM8610 (n = 90, 2 standard deviations (2SD); δ^{202}Hg = -0.54± 0.08‰, Δ^{199}Hg = -0.01 ± 0.05‰, Δ^{200}Hg = 0.01 ± 0.05‰, Δ^{201}Hg = -0.04 ±0.06‰, and Δ^{204}Hg = -0.02 ± 0.10 ‰), which corresponded with previously reported values.[8]

Section V: Calculations for partitioning coefficient (K_d), Bioaccumulation Factor (BAF), and Biomagnification Factor (BMF)

The partitioning coefficient (logK_d) of MeHg transfer from water to particulate (0.7 µm) was calculated according to equation (**Eq. S3**). Where FMeHg (ng L^{-1}) is filtered MeHg, PMeHg (ng L^{-1}) is particulate MeHg, and SPM is the suspended particulate matter (mg L^{-1}).

$$logK_{\ d} = \log\left(\left(\frac{PMeHg}{SPM} * 10^6\right)/FMeHg\right) \qquad \textbf{(Eq. S3)}$$

The bioaccumulation factor (logBAF) was measured to express the phytoplanktonic uptake of MeHg from water and was calculated as the MeHg partitioning from water to the 63-243µm "phytoplankton" fraction (**Eq. S4**). The magnification from the 63-243µm "phytoplankton" to the 243µm "zooplankton" fractions was calculated as the biomagnification factor (BMF, **Eq. S5**).[9]

$$logBAF \ = \log\left((BMeHg_{63-243\mu m} * 1000)/FMeHg\right) \qquad \textbf{(Eq. S4)}$$
$$BMF = BMeHg_{>243\mu m}/BMeHg_{63-243\mu m} \qquad \textbf{(Eq. S5)}$$

All calculated logK_d, logBAF, and BMF values can be found in **Tables S4 and S5**.

Section VI. Mendota and Monona Sediment Core

Elevated THg concentrations in Lake Monona sediments can be observed from depths of 85-58 cm, peaking at 1800 ng g^{-1} at 58 cm (**Figure S2A**), corresponding to historical industrial inputs entering the lake. From 58 to 30 cm, we observe a decline in the sediment THg concentrations in Lake Monona sediments, potentially indicating a decline in Hg effluent from industrial sources. In contrast, Lake Mendota sediment THg concentrations have remained consistent (119.6 ± 64.0 ng/g) through time and 15x lower than the highest THg concentrations observed in Monona. The differences in Hg source histories are also reflected in the δ^{202}THg data of the Mendota and Monona sediment cores (**Figure S2B**). In Lake Monona sediments less negative δ^{202}THg values

(ranging -0.67‰ to -0.38‰, -0.47 ± 0.08‰ average) are observed in the core at the onset of higher Hg concentrations. These values are isotopically enriched when compared to Lake Mendota, which has a consistent δ^{202}THg profile (ranging -1.21‰ to -0.33‰). This suggests that the original contaminant source still persists in lake sediments, indictive of legacy Hg contamination which has been shown to display higher δ^{202}THg values.[10] In contrast, the Lake Mendota sediment δ^{202}THg values are driven more negative from around 50 to 0 cm depth, following a peak in THg concentration and δ^{202}THg, indicating a shift of Hg source to watershed inputs. These watershed inputs are derived from atmospheric Hg deposition onto terrestrial landscapes that enters the watershed via erosion and runoff.[11, 12] Notably, Δ^{199}THg and Δ^{200}THg were both near-zero and comparable throughout the cores for both lakes.[1]

TABLES

Table S1. Quality assurance and checks table for mercury species concentration analyses including recoveries of certified reference materials. Total mercury (THg) and methylmercury (MeHg) concentrations are measured as ng $g^{-1}_{dry\ weight}$. Percent (%) recoveries are calculated as measured value divided by reported value for each certified reference material (CRM). NA indicates not applicable, *n* represents quantity of replicates.

	Purpose	Matrix	*n*	THg	%THg recovered	MeHg	%MeHg recovered
**IAEA-452**	CRM	Scallop tissue	5	146.8±10.7	92±7%	20.6±1.4	94±6%
**IAEA-SL1**	CRM	Sediment	41	134.0±6.8	103±5	NA	NA
**NIST 1944**	CRM	New York/Jersey waterway sediment	24	3826.7±241.1	113±1%	NA	NA
**SQC 1238**	CRM	Sediment	2	NA	NA	10.6±0.0	106±0%
**MSC192AZ**	In-house reference	Seston >243 micron	7	70.3±7.8	NA	49.3±3.5	NA

Table S2. Microwave digest acid test for preparation of total mercury (THg) stable isotopes in seston. All tests were conducted on in-house reference MSC192AZ**,** seston >243 micron collected from Lake Mendota deep hole on 5/18/2021, largely comprised of zooplankton. A variety of acids were tested for complete digestion of seston including nitric acid (HNO_3), hydrochloric acid (HCl), and other acid mixtures listed. Data can be found in associated data release.[1]

Acid Type	Digest recovery (%)	Trap Recovery (%)	δ^{202}THg (‰)	Δ^{199}THg (‰)	Δ^{200}THg (‰)	Notes
Nitric	90 ± 5	90	0.30	0.94	0.15	Dark ring on microwave vessel post-digest
Nitric + Peroxide	94	75	0.76	0.88	0.12	Poor purge and trap recoveries, possible δ^{202}Hg fractionation
Aqua regia (3HCl:1HNO_3)	78 ± 2	NA	NA	NA	NA	Dark ring on microwave vessel post-digest
Reverse aqua regia (1HCl:3HNO_3)	100 ± 5	107 ± 3	0.27 ± 0.01	0.97 ± 0.02	0.12 ± 0.00	Clean microwave vessel post-digest, good purge and trap recoveries

Table S3: Quality assurance and checks table for total mercury (THg) and methylmercury (MeHg) stable isotope analyses including process and instrumental standards as well as certified reference materials (CRM). Standard deviations are reported to 1SD, *n* represents quantity of replicates.

	**Purpose**	**Matrix**	***n***	**δ^{202}THg (‰)**	**Δ^{199}THg (‰)**	**Δ^{200}THg (‰)**	**δ^{202}MeHg (‰)**	**Δ^{199}MeHg (‰)**	**Δ^{200}MeHg (‰)**
**NIST 3133**	Process Standard	NA	48	-0.02±0.11	-0.03±0.05	0.00±0.04	-0.02±0.11	-0.03±0.05	0.00±0.04
**UM Almaden (NIST RM 8610)**	Instrument Standard	NA	90	-0.54±0.04	-0.01±0.03	0.01±0.02	-0.54±0.04	-0.01±0.03	0.01±0.02
**IAEA-452**	CRM	Scallop tissue	6	-0.44±0.05	0.04±0.04	0.03±0.02	-0.60±0.02	0.61±0.04	0.04±0.02
**IAEA-407**	CRM	Fish homogenate	4	0.57±0.03	1.07±0.02	0.05±0.01	NA	NA	NA
**IAEA-SL1**	CRM	Sediment	4	-1.23±0.08	-0.14±0.02	0.04±0.01	NA	NA	NA
**NIST 1944**	CRM	New York/Jersey waterway sediment	1	-0.39	-0.01	0.00	NA	NA	NA
**TORT-3**	CRM	Lobster hepatopancreas	4	NA	NA	NA	0.73±0.06	0.93±0.03	0.07±0.02
**MSC192AZ**	In-house reference	Seston >243 micron	13	0.20±0.07	1.23±0.07	0.13±0.02	0.59±0.12	1.23±0.07	0.13±0.02

Table S4. Calculated seasonal methylmercury (MeHg) bioaccumulation factors (logBAF), biomagnification factors (BMF), partitioning coefficients (logK_d), dissolved organic carbon (DOC), and suspended particulate matter (SPM) concentrations. BMF is the magnification factor from phytoplankton (63-243 micron) to zooplankton (>243 micron), logBAF is the enrichment from water to phytoplankton, and logK_d is the enrichment from aqueous to particulate (depth=0). Calculations can be found in SI Section V. DOC and SPM values can also be found in associated data release.[1]

Lake	Sampling Date	log(BMF) *(Phytoplankton → Zooplankton)*	log(BAF) *(Aqueous → Phytoplankton)*	log(K_d) *(Aqueous → Particulate)*	DOC (mg L^{-1})	SPM (mg L^{-1})
Mendota	4/13/2021	3.44	5.82	5.42	4.58	8.01
	5/18/2021	1.80	6.71	5.80	4.35	0.81
	6/23/2021	5.31	5.61	5.71	4.48	4.39
	7/27/2021	1.72	5.53	5.48	4.62	6.27
	9/10/2021	3.24	5.52	5.57	4.57	5.66
	11/9/2021	3.44	5.43	5.47	4.10	1.83
	2/15/2022	NA	NA	5.65	4.40	0.64
	5/4/2022	2.43	5.93	6.10	4.30	2.94
	6/3/2022	6.22	5.49	5.41	5.08	2.25
	7/12/2022	2.09	5.81	5.63	5.09	4.94
Monona	6/17/2021	5.35	5.92	5.40	4.91	5.75
	8/30/2021	4.44	5.63	6.37	4.88	7.00
	10/27/2021	3.78	5.26	5.58	4.78	3.47
	4/25/2022	3.95	6.03	6.05	5.08	3.93
	6/9/2022	6.79	5.52	5.49	5.74	4.29
	7/14/2022	2.26	5.52	5.29	5.21	6.07

Table S5. Calculated partitioning coefficients (logK_d), dissolved organic carbon (DOC), and suspended particulate matter (SPM) concentration of profile waters collected during stratification. K_d is the enrichment from particulate to water. Calculations can be found in SI Section V. DOC and SPM values can also be found in associated data release.[1]

Lake	Depth (m)	Log(K_d) *(Particulate → Water)*	DOC (mg L^{-1})	SPM (mg L^{-1})
Mendota	0	5.57	4.56	5.66
9/10/21	3	5.66	4.70	4.79
	7.1	5.88	4.65	4.42
	9.5	5.78	4.47	4.29
	10.8	5.34	4.46	4.84
	11.9	4.54	4.45	1.38
	14.6	4.28	4.34	1.22
	16.9	4.50	4.53	0.83
	19.9	3.71	4.49	1.55
Monona	0	5.81	4.91	7.00
8/30/21	2.5	6.08	4.85	8.15
	4.5	6.03	4.65	7.58
	5.8	5.88	4.51	5.48
	8	5.00	4.50	3.58
	9.9	4.98	4.43	1.85

Table S6. Sport fish concentrations and relative source contributions. Source contribution was calculated for each fish as described in the main text according to **Eq.1**, where $f_{epilimnetic}$ and $f_{benthic}$ are the fraction of epilimnetic and benthic-sourced MeHg for each fish. TL indicates total length of each fish. ND indicates not determined. Fish data can be found in previous data release.[13]

Lake	Fish Common Name	Habitat Description	TL (mm)	BTHg (ng g^{-1})	Δ^{199}THg (‰)	$f_{epilimnetic}$	$f_{benthic}$
Mendota	Black Crappie	Pelagic	287	700.0	1.10	87%	13%
	Freshwater Drum	Pelagic	336	837.1	1.04	83%	17%
	Largemouth Bass	Pelagic	349	1050.0	0.97	78%	22%
	Northern Pike	Pelagic	835	3245.7	0.99	80%	20%
	Rock Bass	Pelagic	224	1942.6	1.03	82%	18%
	Walleye	Pelagic	485	1613.0	0.98	79%	21%
	Yellow Perch	Pelagic	222	796.1	0.93	75%	25%
Monona	Largemouth Bass	Pelagic	356	1319.9	1.01	69%	31%
	Largemouth Bass	Pelagic	386.3	666.2	1.00	69%	31%
	Largemouth Bass	Pelagic	316.9	388.1	0.90	61%	39%
	Bluegill	Pelagic	176.9	253.9	0.89	60%	40%
	Bluegill	Pelagic	129.6	305.5	1.01	69%	31%
	Bluegill	Pelagic	ND	165.0	1.07	74%	26%
	Common Carp	Benthic	704	408.8	0.96	ND	ND
	Common Carp	Benthic	610	632.5	0.62	ND	ND
	Common Carp	Benthic	562	780.9	0.80	ND	ND

FIGURES

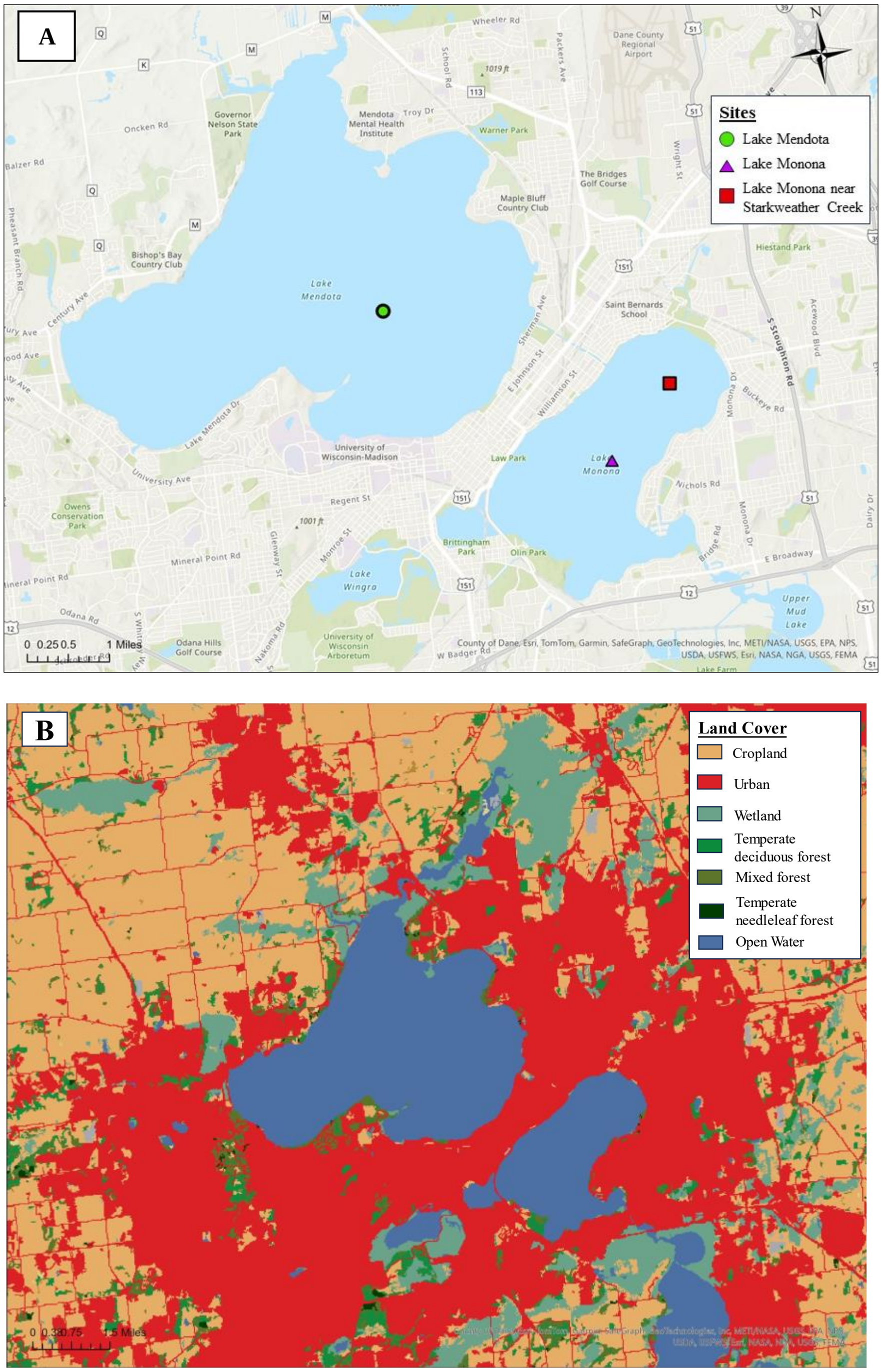

Figure S1: Map of Lakes Mendota and Monona (a) sampling sites and (b) surrounding landcover. Sediment collection sites in panel (a) including Lake Mendota (green circle), Lake Monona (purple triangle), and Lake Monona near Starkweather Creek (red square). This base map image is the intellectual property of Esri used under license. Copyright to Esri and its licensors, all rights reserved. Credit for landcover layer in panel (b) to North American Land Change Monitoring System.[14, 15]

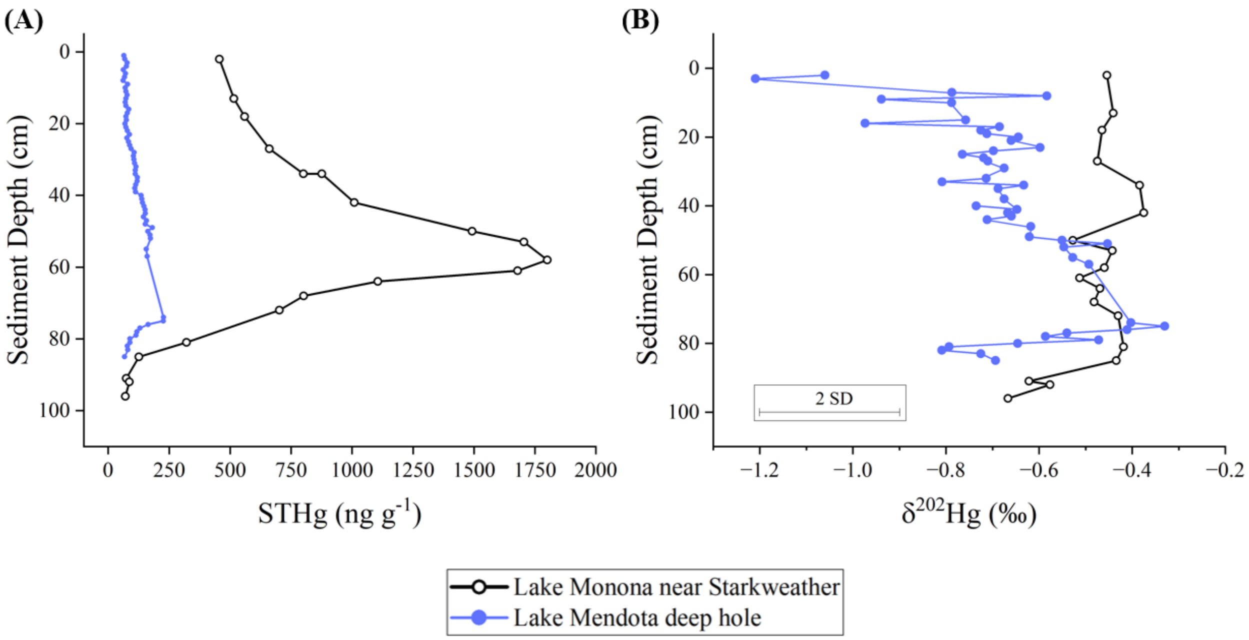

Figure S2. Sediment core mercury profiles from Lakes Mendota and Monona. (a) Total Hg (THg) concentration profiles from Lake Mendota and Monona Starkweather sediment and the associated (b) δ^{202}THg isotopic values of the core. Error bar boxes represent the 2SD of certified reference material IAEA-SL1 (n = 4) of isotopic measurements (**Table S3**). All sediment concentration and isotope values can also be found in associated data release.[1]

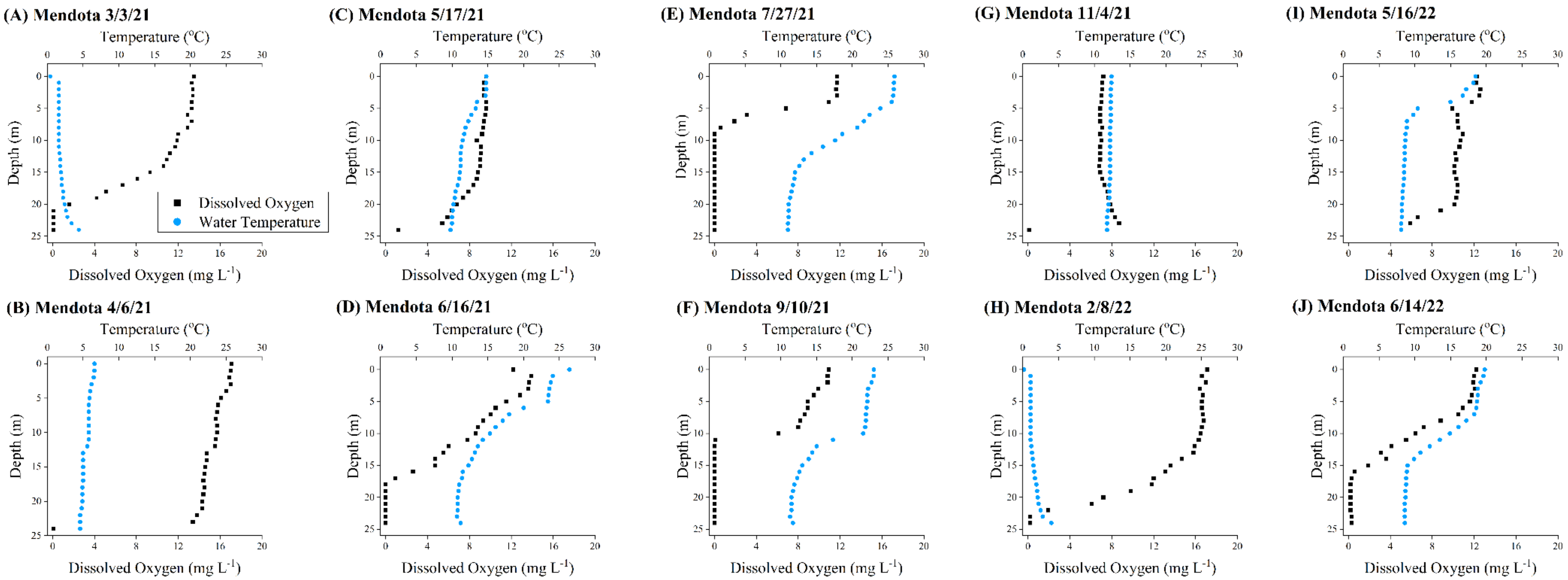

Figure S3. Water Temperature and Dissolved Oxygen profiles for Lake Mendota, roughly corresponding to sampling events in 2021 and 2022. Data were accessed from the North American Temperate Lakes Long-Term Ecological Research (NTL-LTER) portal.

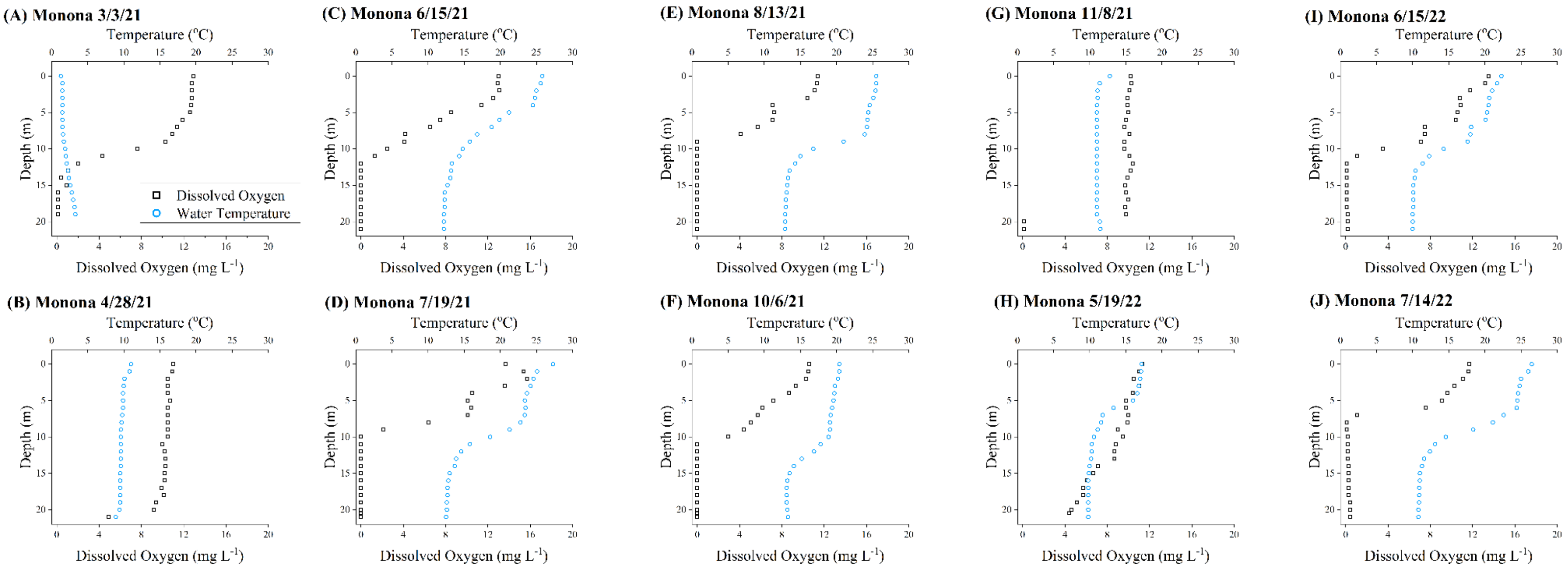

Figure S4. Water Temperature and Dissolved Oxygen profiles for Lake Monona, roughly corresponding to sampling events in 2021 and 2022. Data for these profiles was obtained from the North American Temperate Lakes Long-Term Ecological Research (NTL-LTER) portal.[16]

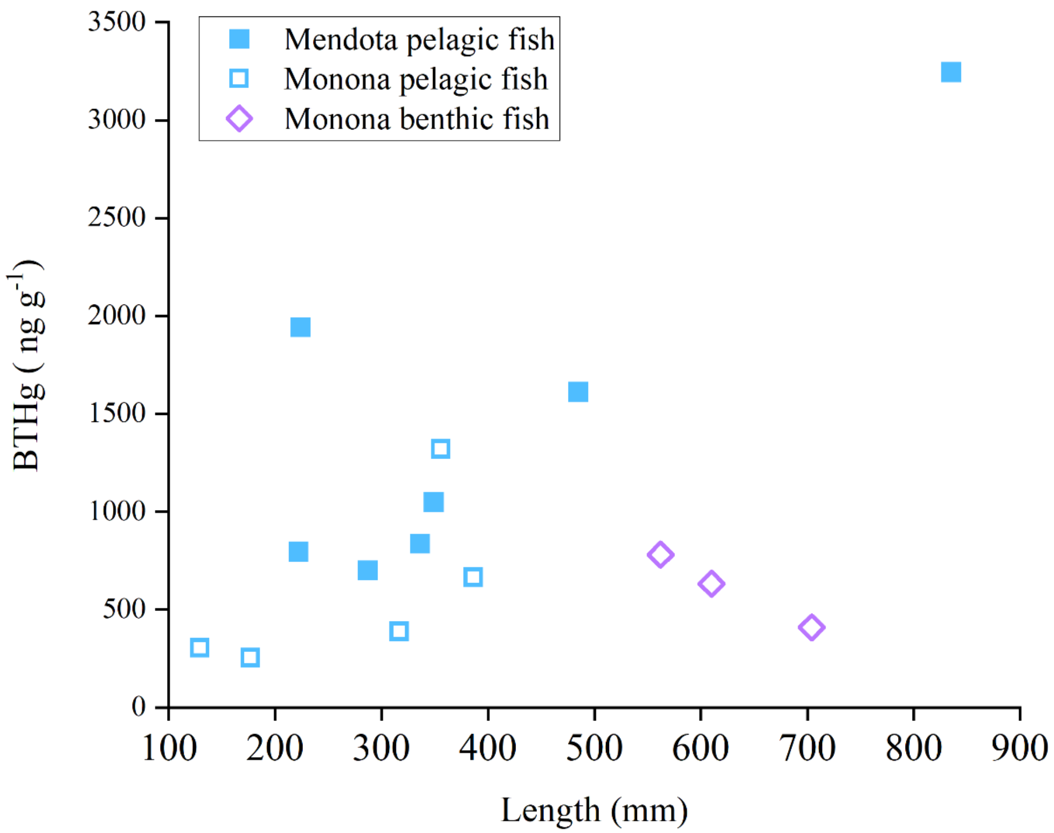

Figure S5. Sport fish biological total mercury (BTHg) versus total fish length. Mendota fish are represented with solid symbols whereas Monona fish have open symbols. Mixed species are presented, broadly separated by habitat type, pelagic (blue, upper waters) and benthic (purple, deeper waters). Fish species can be found in **Table S6**. Data can be found in associated data release.[13]

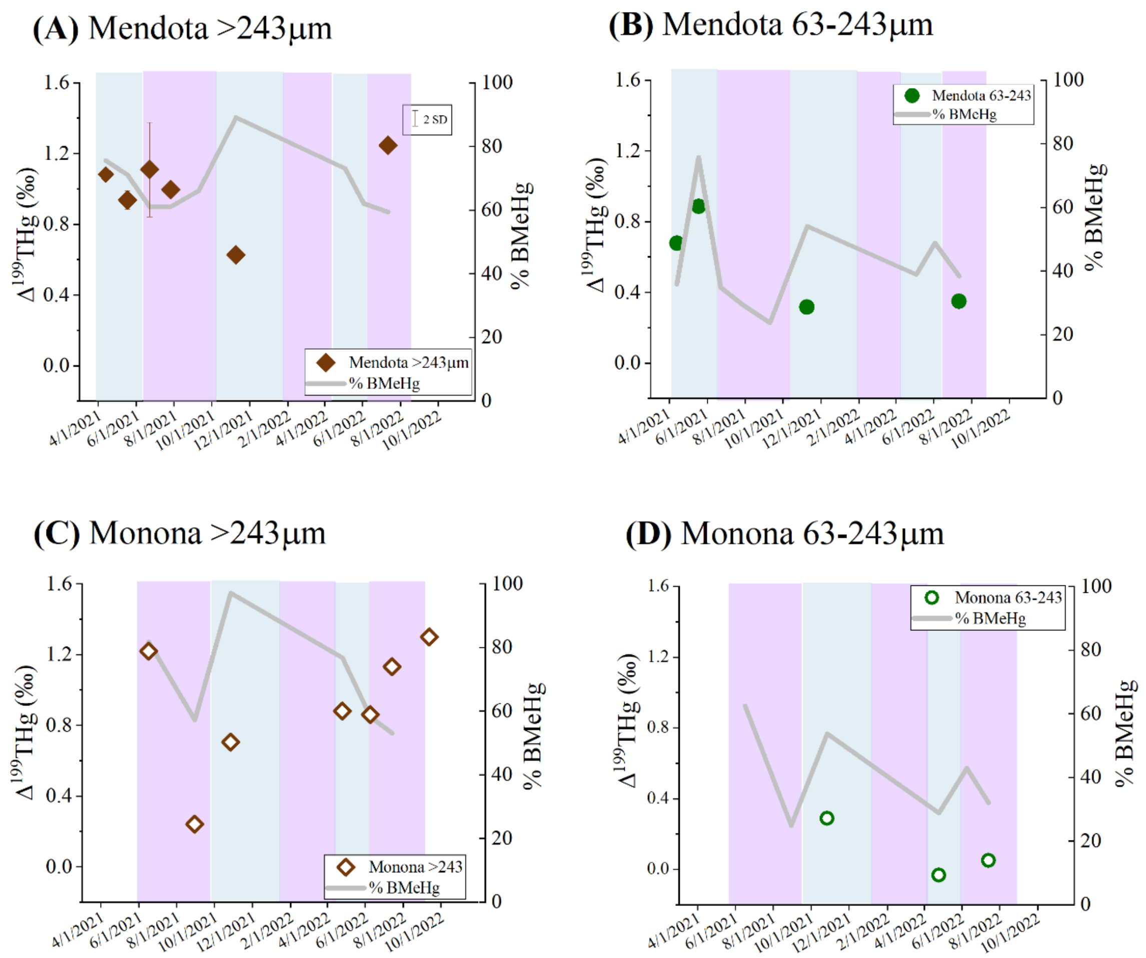

Figure S6. Temporal Δ^{199}THg isotope and percent biological methylmercury (%BMeHg) plots of seston fractions in (A) Lake Mendota and (B) Lake Monona. Seston fractions were 63-243 µm ~ "phytoplankton", >243 µm ~ "zooplankton". Error bars on points represent ±1SD of sample replicates. 2SD box represents 2SD of Δ^{199}THg values for IAEA-452 (n = 6). Additional isotopic plots (δ^{202}THg and Δ^{200}THg) can be found in **Fig. S9**. Colored bars represent lake water conditions: mixed (blue) and stratified (purple) as determined from the North American Temperate Lakes Long-Term Ecological Research (NTL-LTER) water temperature and dissolved oxygen profiles (**Fig. S3, S4**).[16] Isotope data can be found in associated data release.[1]

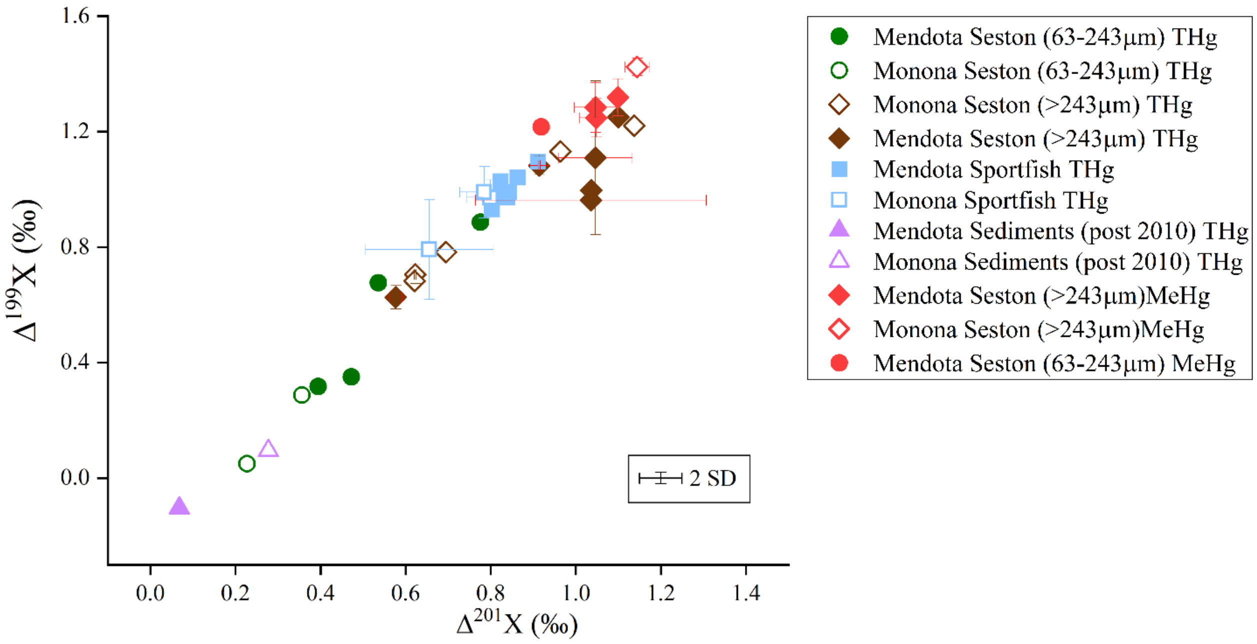

Figure S7. Photochemical slope biplot of Δ^{199}X versus Δ^{201}X isotopic composition, where X is mercury species: total mercury (THg) or methylmercury (MeHg), of sediments, seston (63-243µm ~ "phytoplankton", >243µm ~ "zooplankton"), and fish from both lakes. The error box represents the 2SD of certified reference material IAEA-407 measurements of isotopic ratios of Δ^{199}Hg and Δ^{201}Hg and error bars represent ±1SD of sample replicates or composites. Isotopic data can be found in associated data releases.[1,13]

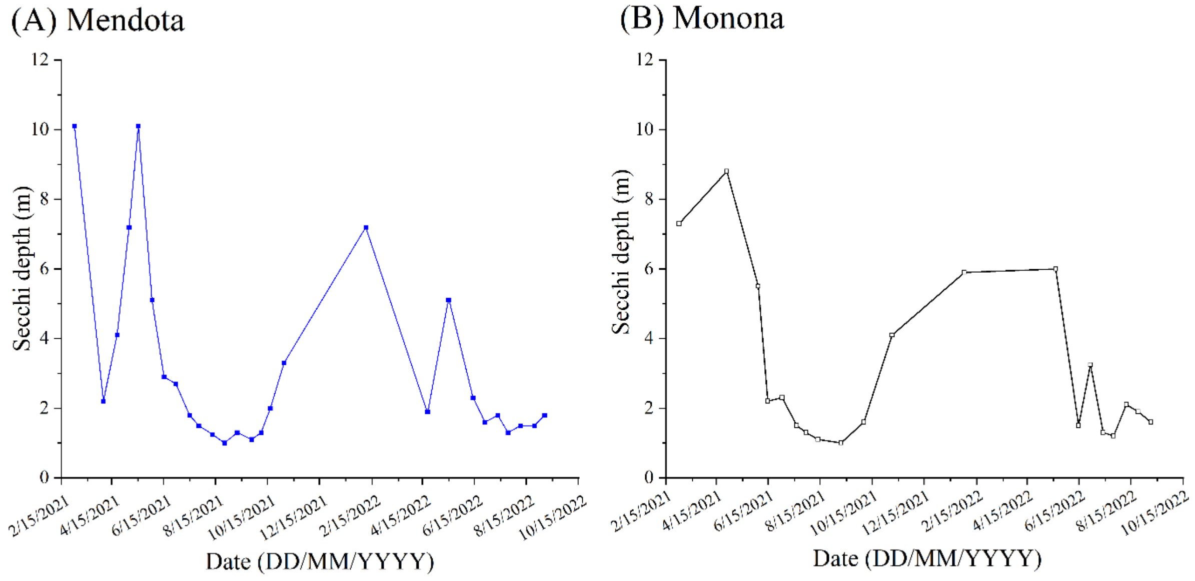

Figure S8. Secchi depth, a measurement of water clarity, for (A) Lake Mendota and (B) Lake Monona, roughly corresponding to sampling events in 2021 and 2022. Data for these profiles was obtained from the North American Temperate Lakes Long-Term Ecological Research (NTL-LTER) portal.[17]

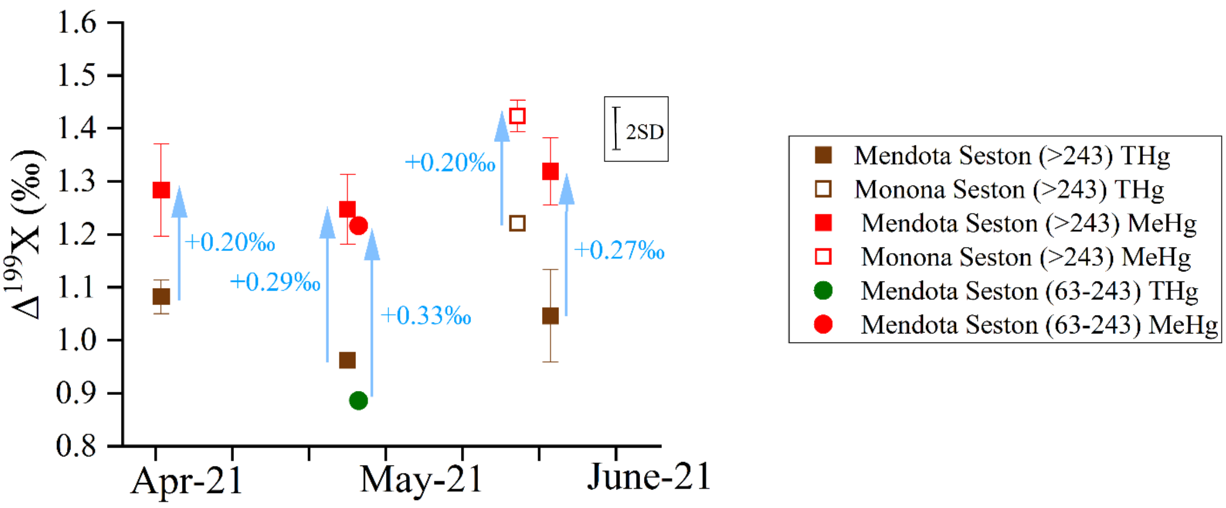

Figure S9. THg and MeHg Δ^{199}X comparison plots for >243 and 63-243µm seston fractions. (A) biplot of Δ^{199}X vs. δ^{202}X (X is mercury species: total mercury (THg) or methylmercury (MeHg; red symbols) isotopic signatures of seston from both lakes. 2SD box represents 2SD of Δ^{199}Hg values for IAEA-452 (n = 6). Isotope data can be found in associated data release.[1]

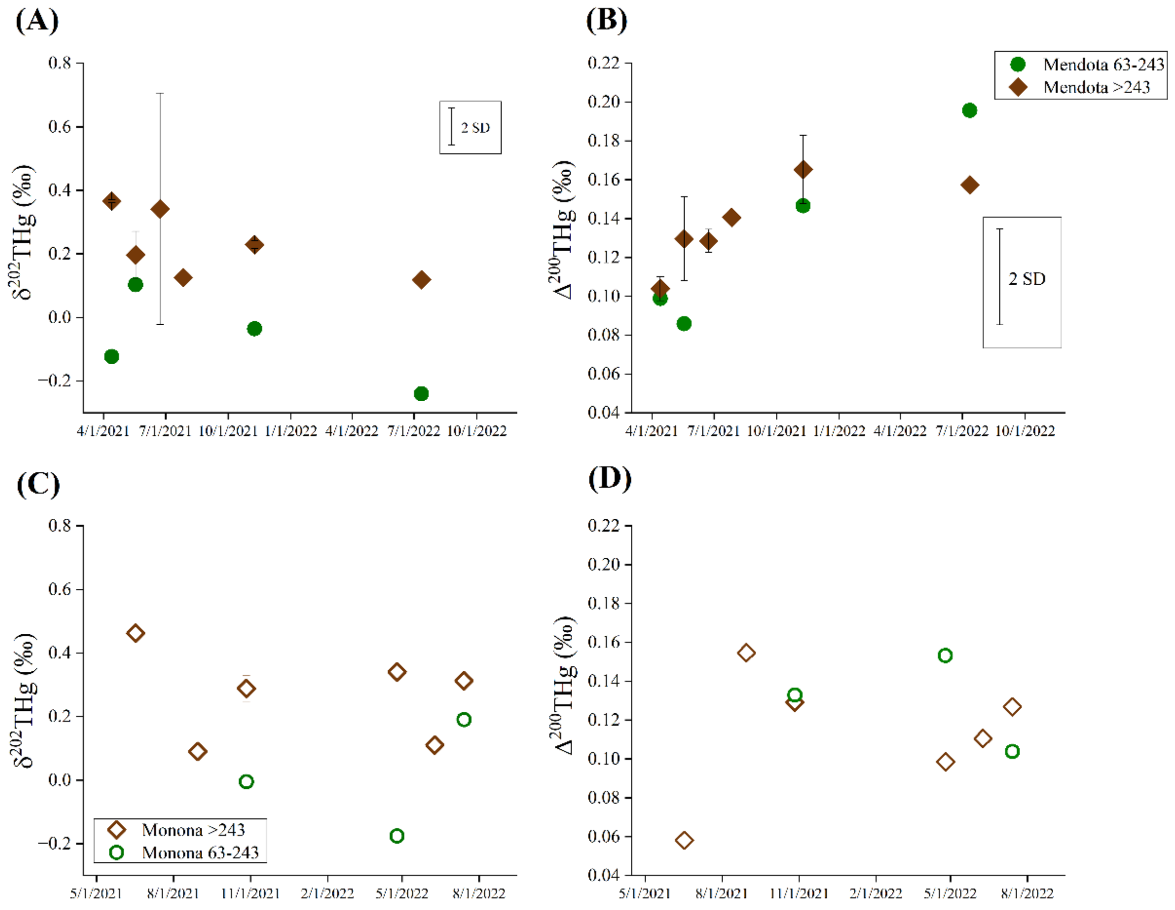

Figure S10. Temporal δ^{202}THg and Δ^{200}THg stable isotope plots of plankton fractions in Lake Mendota (A, B) and Lake Monona (C, D). Error bars on points represent 1 SD of sample replicates. 2SD box represents 2SD of δ^{202}Hg or Δ^{200}Hg values for IAEA-452 (n = 6). Isotope data can be found in associated data release.[1]

REFERENCES

(1) Armstrong, G. J., Janssen, S. E., Tate, M. T., Rosera, T. J., Lepak, Ryan., F. Temporal Mercury Concentration and Stable Isotope Assessment of Seston, Waters, Sediment, and Particulates from Lakes Mendota and Monona, Madison, Wisconsin, 2021-2022: U.S. Geological Survey Data Release. 2024.

(2) Rosera, T. J.; Janssen, S. E.; Tate, M. T.; Lepak, R. F.; Ogorek, J. M.; DeWild, J. F.; Krabbenhoft, D. P.; Hurley, J. P. Methylmercury Stable Isotopes: New Insights on Assessing Aquatic Food Web Bioaccumulation in Legacy Impacted Regions. *ACS ES&T Water* **2022**. DOI: 10.1021/acsestwater.1c00285.

(3) Lepak, R. F.; Janssen, S. E.; Yin, R.; Krabbenhoft, D. P.; Ogorek, J. M.; Dewild, J. F.; Tate, M. T.; Holsen, T. M.; Hurley, J. P. Factors Affecting Mercury Stable Isotopic Distribution in Piscivorous Fish of the Laurentian Great Lakes. *Environmental Science and Technology* **2018**, *52* (5), 2768-2776. DOI: 10.1021/acs.est.7b06120.

(4) Janssen, S. E.; Lepak, R. F.; Tate, M. T.; Ogorek, J. M.; DeWild, J. F.; Babiarz, C. L.; Hurley, J. P.; Krabbenhoft, D. P. Rapid Pre-concentration of Mercury in Solids and Water for Isotopic Analysis. *Analytica Chimica Acta* **2019**, *1054*, 95-103. DOI: 10.1016/j.aca.2018.12.026.

(5) Agency, U. S. E. P. Method 1630: Methylmercury in Water by Distillation, Aqueous Ethylation, Purge and Trap, and Cold Vapor Atomic Fluorescence Spectrometry. US Environmental Protection Agency Washington, DC: 1998.

(6) Rosera, T. J.; Janssen, S. E.; Tate, M. T.; Lepak, R. F.; Ogorek, J. M.; DeWild, J. F.; Babiarz, C. L.; Krabbenhoft, D. P.; Hurley, J. P. Isolation of methylmercury using distillation and anion-exchange chromatography for isotopic analyses in natural matrices. *Analytical and Bioanalytical Chemistry* **2020**, *412* (3), 681-690. DOI: 10.1007/s00216-019-02277-0.

(7) Blum, J. D.; Bergquist, B. A. Reporting of Variations in the Natural Isotopic Composition of Mercury. *Analytical and Bioanalytical Chemistry* **2007**, *388* (2), 353-359. DOI: 10.1007/s00216-007-1236-9.

(8) Blum, J. D.; Johnson, M. W. Recent developments in mercury stable isotope analysis. *Reviews in Mineralogy and Geochemistry* **2017**, *82* (1), 733-757.

(9) Ogorek, J. M.; Lepak, R. F.; Hoffman, J. C.; DeWild, J. F.; Rosera, T. J.; Tate, M. T.; Hurley, J. P.; Krabbenhoft, D. P. Enhanced Susceptibility of Methylmercury Bioaccumulation into Seston of the Laurentian Great Lakes. *Environmental Science & Technology* **2021**, *55* (18), 12714-12723. DOI: 10.1021/acs.est.1c02319.

(10) Eckley, C. S.; Gilmour, C. C.; Janssen, S.; Luxton, T. P.; Randall, P. M.; Whalin, L.; Austin, C. The assessment and remediation of mercury contaminated sites: A review of current approaches. *Sci Total Environ* **2020**, *707*, 136031. DOI: 10.1016/j.scitotenv.2019.136031.

(11) Janssen, S. E.; Hoffman, J. C.; Lepak, R. F.; Krabbenhoft, D. P.; Walters, D.; Eagles-Smith, C. A.; Peterson, G.; Ogorek, J. M.; DeWild, J. F.; Cotter, A.; et al. Examining historical mercury sources in the Saint Louis River estuary: How legacy contamination influences biological mercury levels in Great Lakes coastal regions. *Science of The Total Environment* **2021**, *779*, 146284. DOI: https://doi.org/10.1016/j.scitotenv.2021.146284.

(12) Lepak, R. F.; Yin, R.; Krabbenhoft, D. P.; Ogorek, J. M.; DeWild, J. F.; Holsen, T. M.; Hurley, J. P. Use of Stable Isotope Signatures to Determine Mercury Sources in the Great Lakes. *Environmental Science & Technology Letters* **2015**, *2* (12), 335-341. DOI: 10.1021/acs.estlett.5b00277.

(13) Janssen, S. E., and Lepak, R.F. Mercury Stable Isotope Assessment of Global Food Webs: U.S. Geological Survey data release. 2023.

(14) Yang, L.; Jin, S.; Danielson, P.; Homer, C.; Gass, L.; Bender, S. M.; Case, A.; Costello, C.; Dewitz, J.; Fry, J. A new generation of the United States National Land Cover Database: Requirements, research priorities, design, and implementation strategies. *ISPRS journal of photogrammetry and remote sensing* **2018**, *146*, 108-123.
(15) Survey, U. S. G. National Land Cover Database (NLCD) 2019 Land Cover Conterminous United States. The NLCD product is the version dated June 4, 2021. U.S. Geological Survey; Sioux Falls, South Dakota, USA, 2021.
(16) Magnuson, J. J., S.R. Carpenter, and E.H. Stanley. North Temperate Lakes LTER: Physical Limnology of Primary Study Lakes 1981 - current ver 35. Initiative, E. D., Ed.; 2023.
(17) Magnuson, J. J., S.R. Carpenter, and E.H. Stanley. North Temperate Lakes LTER: Secchi Disk Depth; Other Auxiliary Base Crew Sample Data 1981 - current ver 32. Initiative, E. D., Ed.; 2023.
